# Removal of VirD2 from a synthetic T-DNA complex of *Agrobacterium* by the host DNA repair protein TDP2

**DOI:** 10.1371/journal.ppat.1014750

**Published:** 2026-10-26

**Authors:** Benoît Lacroix, Vitaly Citovsky

**Affiliations:** Department of Biochemistry and Cell Biology, Stony Brook University, Stony Brook, New York, United States of America; The Ohio State University, UNITED STATES OF AMERICA

## Abstract

During plant genetic transformation, *Agrobacterium* transfers to its host cell a single-stranded segment of DNA (T-DNA), as a covalent complex with the virulence protein VirD2, which is ultimately integrated into the plant genome. The covalently bound VirD2 molecule is an obstacle to the ligation of the T-DNA 5’-end to host genomic DNA; therefore, VirD2 must be cleaved from the VirD2-T-DNA complex (T-complex) before the integration is completed. An interesting idea was raised that TDP2 (tyrosine phosphodiesterase 2), a protein known to resolve protein-DNA adducts, could represent a pathway for VirD2 removal from the T-complex; however, this function has not been proven directly. Here, we developed a simple technique to generate fluorescently labeled VirD2-ssDNA complexes *in vitro* and explored the DNA substrate requirements for the VirD2-mediated formation of these complexes, establishing a tractable experimental system for studying VirD2 removal from synthetic T-complexes. Using this system, we demonstrated that TDP2, from mammalian or plant origin, can specifically cleave VirD2 from these synthetic T-complexes; this activity was Mg^2+^-dependent and required a conserved C-terminal histidine residue of TDP2. Our results strongly support the hypothesis that plant TDP2 can remove VirD2 from the T-DNA before its integration into the plant genome.

## Author summary

Agrobacterium tumefaciens and related species are fascinating pathogenic bacteria that can genetically transform plants by transferring and integrating a segment of their own DNA into the genome of their host cells. This extraordinary ability has been harnessed as an essential tool for research and biotechnology. The transferred DNA (T-DNA) is exported from the bacterial cell to its host cell nucleus as a complex (named the T-complex) comprised of a single-stranded DNA segment covalently attached to the effector protein VirD2; it is thus necessary that VirD2 is cleaved from the T-DNA to allow its ligation to the genome DNA. Here, we generated fluorescently labeled artificial T-complexes and developed a system suitable for in vitro study of VirD2 removal from T-complex. Next, we demonstrated that purified TDP2 is indeed able to cleave precisely VirD2 from the imported T-DNA strand. Our findings provide new insights into the mechanism of Agrobacterium T-complex processing prior to T-DNA integration, and highlight how the activity of host plant factors can be recruited to facilitate Agrobacterium infection process.

## Introduction

*Agrobacterium tumefaciens* is a plant pathogenic bacterium known to transfer a single-stranded segment (T-strand) of its tumor-inducing (Ti) plasmid DNA (T-DNA) to its host cell genome, which causes the crown gall disease in plants and is also widely used as a tool for plant and fungal genetic modification [1,2]. Whereas the integration of *Agrobacterium* T-DNA into the host genomic DNA represents a crucial step of the infection process, its molecular mechanisms are not fully understood [3,4]. Double-stranded DNA breaks (DSBs) in the host cell genome have been shown to act as preferential targets for T-DNA integration [5,6], logically implying that DNA repair is involved in integration. Indeed, *Agrobacterium* T-DNA integration is known to rely mostly on the host DNA repair pathways. For example, using *Saccharomyces cerevisiae* as a host, the T-DNA integration outcome of *Agrobacterium* infection was strictly dependent on the host DNA repair pathways [7,8]. In addition, the expression of plant genes involved in DNA damage response and repair was induced upon inoculation of virulent *Agrobacterium* [9,10]. In plants, initial studies using *Arabidopsis* mutants in different components of the most common DNA repair pathways were not conclusive, with different studies suggesting that the same factors were either required or not required for integration, e.g.[11,12],. Systematic studies using mutants in the main DNA repair pathways suggested that none of these pathways were individually required, while plants mutated in several of these pathways showed reduced T-DNA integration rates [13,14]. These apparent contradictions could partially result from differences in transformation protocols, such as floral dip, root transformation, and different bacterial inocula, but they most likely reflect several concurrent T-DNA integration pathways, which is consistent with the functional redundancy of the different DNA repair pathways [14,15].

Recently, DNA polymerase theta (Pol θ)-mediated DNA repair (TMEJ, Theta-Mediated End-Joining) has emerged as one of the major pathways for T-DNA integration; indeed, signature sequences of Pol θ activity were found at the T-DNA-plant genome junctions, and T-DNA integration was inhibited in an *Arabidopsis* Pol θ mutant [16]. The occurrence of Pol θ activity footprint at T-DNA integration sites was later confirmed in several other plant species [17]. In other reports, *Arabidopsis* mutants in Pol θ were not completely resistant to *Agrobacterium*-mediated genetic transformation [14,18], but transformation levels were significantly reduced, consistent with TMEJ being the major, albeit not unique, pathway for T-DNA integration. In the TMEJ pathway, also known as microhomology end-joining (MMEJ) or alternative non-homologous end-joining (alt-NHEJ), short-sequence homologies between single-stranded DNA at each end of a break anneal before second-strand DNA synthesis mediated by Pol θ and ligation mediated by the TMEJ or classical NHEJ pathway [19,20]; interestingly, in mammalian cells, inactivation of both pathways blocked random integration of exogenous DNA [21,22]. In the current model for the TMEJ-mediated T-DNA integration, the 3’-end of the T-DNA is first captured at genomic DNA break sites by Pol θ, before the second strand is synthesized by Pol θ and integration is completed by ligation of the 5’-end of the T-strand with genomic DNA [16].

The T-strand is translocated to its host cell as a nucleoprotein complex, T-complex, formed in the *Agrobacterium* cell by the endonuclease VirD2 that remains covalently bound to the 5’-end of the T-strand [23]. This VirD2 molecule represents a physical obstacle to ligation of the T-strand 5’-end to the host genomic DNA; therefore, dissociation of VirD2 from the T-DNA is a necessary step before the T-DNA integration is completed. Mutational analysis of Arabidopsis plants has shown that two pathways exist for the removal of VirD2 from the T-complex [24]. First, resection of VirD2 bound to the T-strand can occur via the MRN (MRE11-RAD50-NBS1) pathway. The MRN protein complex plays essential roles in DNA damage response and DSB repair, such as DNA binding and processing, via the nuclease activity of MRE11, as well as recruiting other proteins involved in DNA repair [25]. Alternatively, cleavage of VirD2 from its complex with the T-strand can be effected by the tyrosyl-DNA phosphodiesterase 2 enzyme (TDP2) [24,26], known to remove DNA-protein crosslinks occurring during genomic DNA replication and transcription, specifically in cleavage of tyrosyl-DNA bonds that are formed in topoisomerase 2 adducts [27–29].

Here, we focused on the putative TDP2-based pathway for VirD2 removal. First, we developed a simple method to generate synthetic VirD2-ssDNA T-complexes and explored the DNA substrate requirements for the VirD2-mediated formation of these complexes, establishing a tractable experimental system for studying VirD2 removal from the synthetic T-complexes *in vitro*. Using this system, we demonstrated that recombinant TDP2, both from mammal and plant origins, can specifically cleave VirD2 from these synthetic T-complexes.

## Results

### Generation of fluorescent synthetic T-complexes

Previously, VirD2-ssDNA complexes have been generated using ssDNA substrates radioactively labeled at their 3’-end [30–32], whereas ssDNA substrates tagged with a fluorescent marker Cy5 were reported to form complexes with a truncated VirD2 protein [33]. In a conceptual combination of these approaches, we used synthetic ssDNA segments of different lengths, carrying a right T-DNA border (RB) sequence specifically recognized and cleaved by VirD2 [34] and labeled at their 3’-end with a 5’-fluorescein phosphoramidite (FAM), and the full-length recombinant VirD2 protein. Incubation of purified VirD2, tagged with T7 and 6xHis epitopes (the term VirD2 in all the subsequent experiments refers to this tagged protein), with fluorescently labeled ssDNA is expected to result in a fluorescently tagged complex composed of VirD2 covalently attached to the 5’ end of ssDNA and a short unlabeled ssDNA segment liberated following ssDNA cleavage by VirD2; because the reaction is incomplete, the reaction products are also expected to contain the residual free VirD2 and free fluorescent ssDNA. (Fig 1A). We reacted the wild-type C58 VirD2 with a 36-nt fluorescently labeled oligomer, corresponding to a C58 Ti-plasmid sequence containing the RB border and 9 nt downstream of the cleavage site [30,32] (S1 Table), resolved the products by SDS-PAGE, and visualized them by fluorescence imaging followed by Coomassie blue staining. In control experiments, we utilized the VirD2 Y29A mutant with its endonuclease/nicking activity abolished by replacing tyrosine 29, which forms the phosphodiester bond with ssDNA, with alanine [35]. Fig 1B shows that a fluorescent VirD2-ssDNA complex was formed with the wild-type VirD2, but not with the Y29A mutant; this complex was detected by fluorescence imaging as a band with electrophoretic mobility slightly slower than the 72 kDa size marker, while free fluorescent ssDNA substrate was visible at the migration front of the gel (top panel). Protein staining of the same gel (Fig 1B, bottom panel) detected a band with a ca.74 kDa apparent mass, corresponding to the fluorescent synthetic T-complex observed in the fluorescence image (Fig 1B, top panel), whereas free VirD2 with an apparent mass of ca.70 kDa was observed in all lanes. We detected no significant differences in the formation of the synthetic T-complex at 25°C and 37°C and reaction times of 1 h and 3 h (Fig 1C). After incubation, the mixture also contained unreacted fluorescently labeled ssDNA and free VirD2, consistent with the reversible nature of this reaction [30].

**Fig 1:**
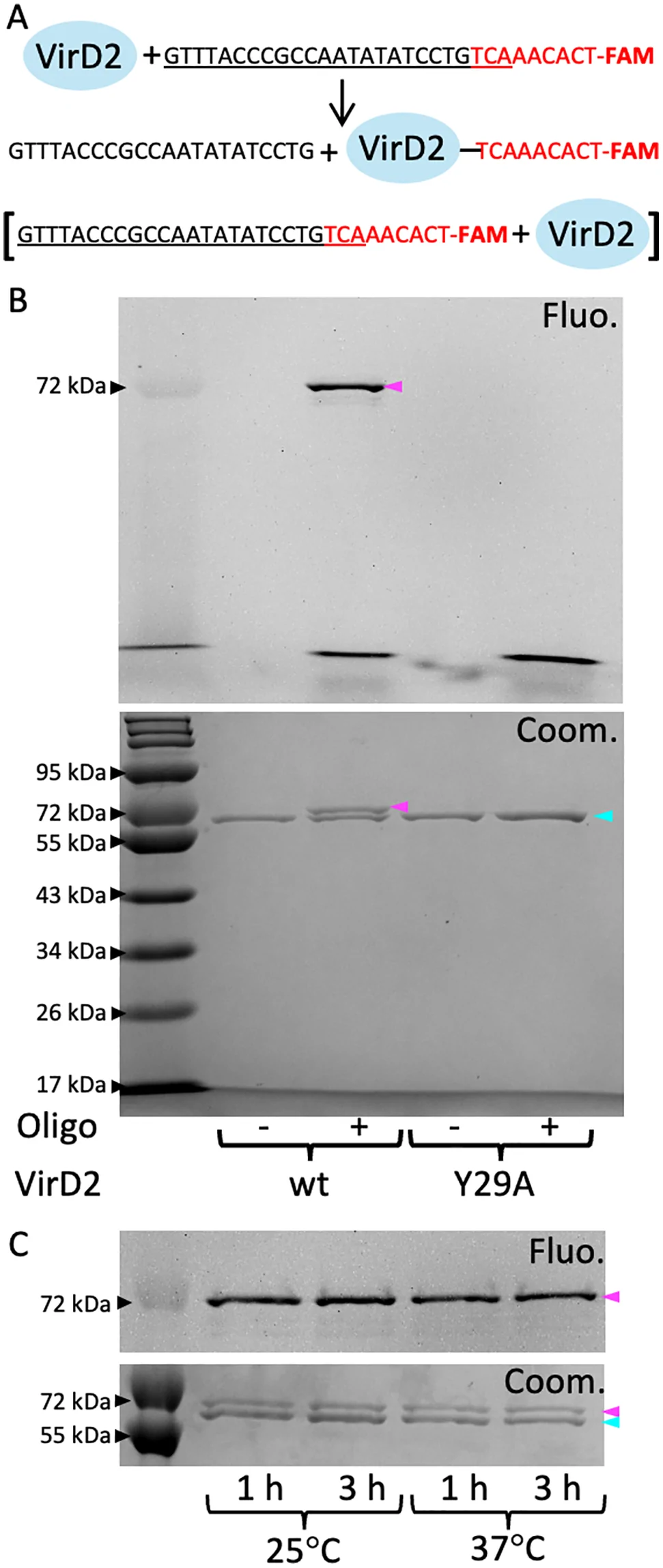
Generation of fluorescently labeled VirD2-ssDNA complexes. **(A)** Schematic illustration of the reaction between VirD2 and a ssDNA substrate. Incubation of purified VirD2 and an ssDNA oligomer containing the T-DNA right border (RB) sequence of the Ti-plasmid of the *A. tumefaciens* C58 strain and fluorescently tagged with FAM results in cleavage at RB and generation of a VirD2-ssDNA complex. The 25-bp sequence corresponding to the T-DNA right border (RB) is underlined, and sequences that remain attached to VirD2 in the VirD2-ssDNA complex are indicated in red. **(B)** Electrophoretic analysis of VirD2-ssDNA complex formation. The reaction mixtures were resolved by SDS-PAGE on a 10% gel, and free VirD2 and VirD2-ssDNA complexes were visualized by fluorescence imaging (Fluo., top panel) and Coomassie blue staining (Coom., bottom panel). **(C)** Effects of temperature and incubation time on VirD2-ssDNA complex formation. The 72-kDa size marker is indicated by a black arrowhead; VirD2 and VirD2-ssDNA complexes are indicated by cyan and magenta arrowheads, respectively; wt, wild-type. Note that the 72-kDa size marker is weakly fluorescent, providing a useful size indicator for fluorescence observations.

### Effects of ssDNA substrate length on the formation of synthetic T-complexes

To identify the shortest sequence sufficient to be recognized by VirD2 for cleavage and subsequent T-complex formation, we examined a series of ssDNA substrates with decreasing length starting from the 36-nt segment (Fig 1A), from the 5’ end of which nucleotides were progressively removed (Fig 2A). Based on the fluorescence imaging and protein staining analyses, VirD2 was still active on these substrates down to the 15-nt segment, containing only 6 nt before the VirD2 cleavage site (ATCCTG, Fig 2A). Interestingly, the efficiency of the reaction increased from the longer to the shorter of these sequences; the intensities of both the fluorescently labeled and protein-stained T-complex-specific bands increased, while the lower protein-stained band, corresponding to free VirD2, decreased. We further analyzed the 15-nt segment by progressive 3’-end deletions. The minimal sequence recognized and processed by VirD2, although with a lower efficiency, was an 8-nt segment containing 6 nt upstream and 2 nt downstream of the cleavage site; no T-complex formation was observed with shorter segments (Fig 2B). Note that for VirD2-ssDNA complexes with 2–3 nt remaining attached to VirD2, free VirD2 and VirD2-ssDNA cannot be resolved under our electrophoresis conditions and appear as a single Coomassie blue-stained band (Fig 2B). Finally, we generated VirD2 complexes with longer fluorescently labeled ssDNA substrates, reaching up to a 94-nt segment covalently attached to VirD2; with this longest segment, the unreacted 100-nt ssDNA substrate is detected as a fluorescent band of ca. 30 kDa (Fig 2C).

**Fig 2:**
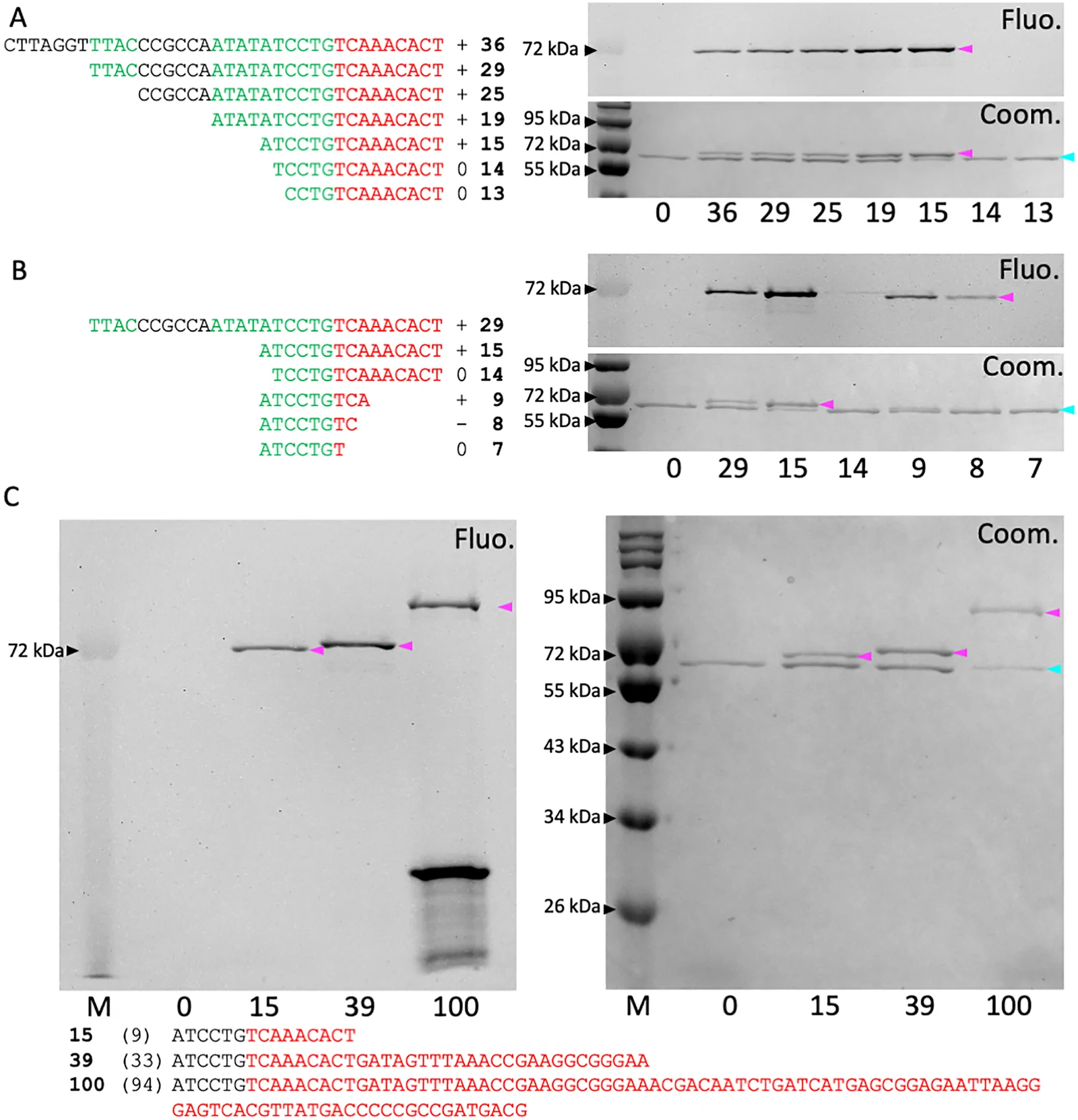
Synthetic T-complexes with different lengths of ssDNA. **(A)** Reducing the length of the ssDNA substrate from its 5’-end. “+”, “-“, and “0” indicate, respectively, efficient, weak, or absent formation of the VirD2-ssDNA complex. **(B)** Reducing the length of the ssDNA substrate from its 3’-end. Each lane is labeled with the total number of nt in the ssDNA substrate; the two highly conserved sequences within the 25-bp of the right border are indicated in green; sequences that remained attached to VirD2 in the VirD2-ssDNA complex are indicated in red. **(C)** Increasing the length of the ssDNA substrate. Each lane is labeled with the total number of nt in the ssDNA substrate; numbers of nt that remain attached to VirD2 are in parentheses; VirD2 and VirD2-ssDNA complexes are indicated by cyan and magenta arrowheads, respectively.

### Removal of VirD2 from a synthetic T-complex by human TDP2

We then examined whether TDP2 can disassemble the synthetic T-complex by severing VirD2 from ssDNA. We selected a 22-nt ssDNA substrate with 6 nt upstream of the cleavage site for generation of a T-complex with a 16-nt ssDNA attached to a VirD2 molecule. Experiments in Fig 2 showed that generation of the VirD2-ssDNA complex was maximally efficient with 6 nt upstream of the cleavage site and that complexes with different lengths of ssDNA segment attached to VirD2 could be obtained. The size of a complex with a 16 nt ssDNA segment allowed its easy electrophoretic distinction from free VirD2. The flowchart for the experimental procedure for preparation of the synthetic T-complex and detection of VirD2 removal by TDP2 is shown in S1 Fig. Briefly, the fluorescent VirD2-ssDNA complex was produced, concentrated by centrifugation, and incubated with TDP2; initially, we took advantage of the commercially available human TDP2 (HsTDP2, harboring GST and 6-His tags). For protein and nucleoprotein complex detection, samples were then separated on 10% SDS-polyacrylamide gels and analyzed by fluorescence imaging and western blotting using anti-His antibody for simultaneous detection of both VirD2 and TDP2 tagged with the 6xHis epitope. For detection of ssDNA, samples were separated on 15% TBE-Urea gels and analyzed by fluorescence imaging.

Fig 3A shows that our Western blot analysis can separate free VirD2, VirD2-ssDNA complexes, and HsTDP2, and identify them by their size on the same blot; note that although HsTDP2 is smaller than VirD2, the commercial HsTDP2 displays a larger apparent mass because it is also fused with a 26-kDa glutathione S-transferase (GST) tag. We then incubated the fluorescent VirD2-ssDNA complexes for 45 min at 37°C with increasing concentrations of HsTDP2, in the presence or absence of MgCl_2_. Fluorescence imaging revealed that, in the presence of MgCl_2_, HsTDP2 promotes removal of VirD2 attached to ssDNA, as indicated by gradual disappearance of the T-complex (Fig 3, top and middle panels) and appearance of free 16-nt ssDNA (Fig 3, bottom panel). Consistently, Western blotting detected reduced amounts of the T-complex and increased amounts of free VirD2 and HsTDP2 (Fig 3, middle panel). No significant disassembly of VirD2-ssDNA was observed in the absence of MgCl_2_, consistent with the known absolute requirement for magnesium ions for the TDP2 enzymatic activity [36]. That we detected no ssDNA species other than 22 nt, corresponding to the unreacted ssDNA substrate, or 16 nt, corresponding to ssDNA liberated from the T-complex (Fig 3B, bottom panel), suggests that HsTDP2 did not cleave the phosphodiester bonds within ssDNA, and that the cleavage occurred between the VirD2 Tyr-29 residue and the first nucleotide of the ssDNA segment attached to VirD2. Collectively, these data suggest that HsTDP2 can remove the VirD2 covalently attached to the synthetic *Agrobacterium* T-complex.

**Fig 3:**
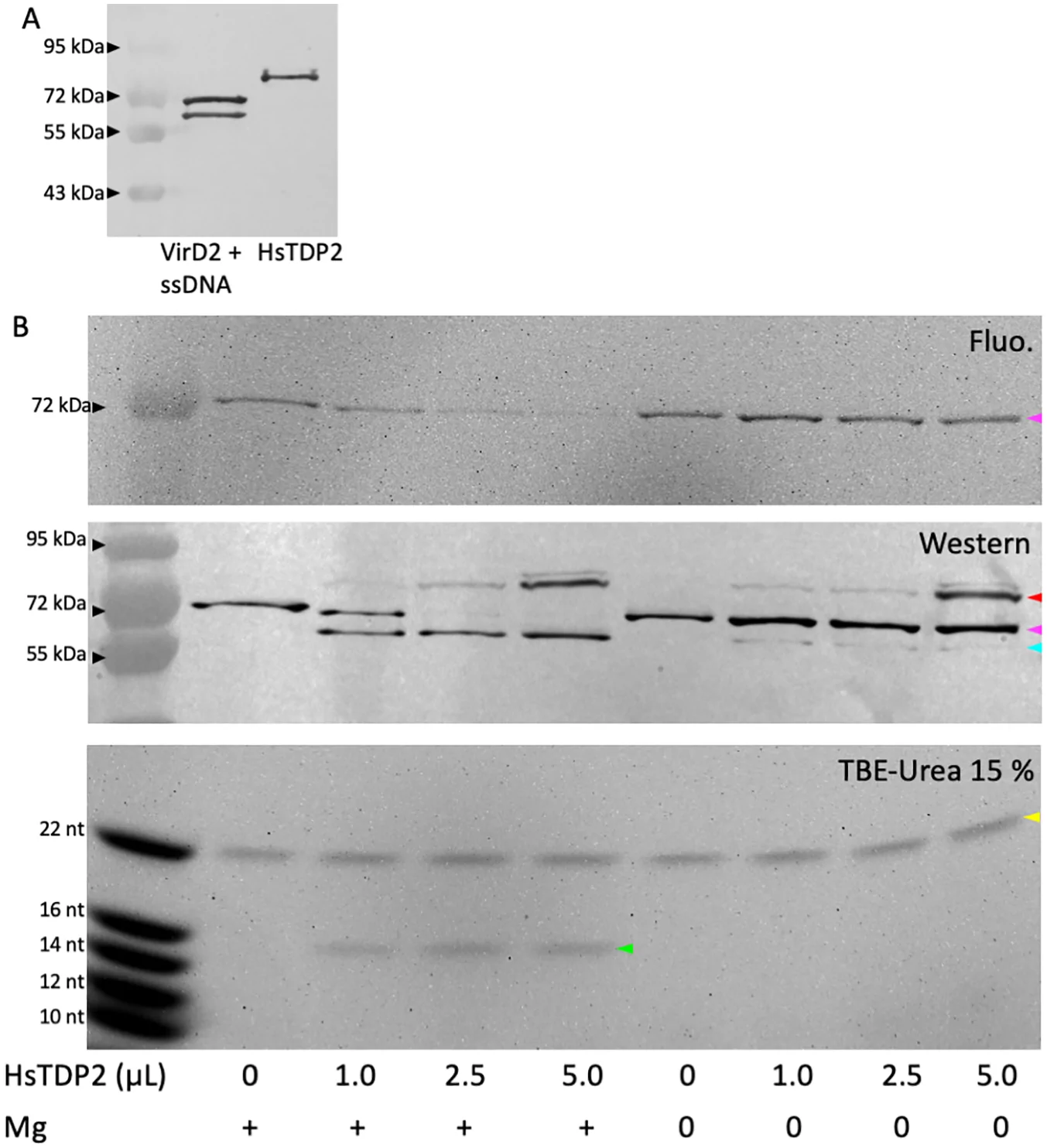
Removal of VirD2 from the synthetic T-complex by HsTDP2. **(A)** Western blot detection of VirD2-ssDNA and HsTDP2 using anti-6xHis antibodies. **(B)** Electrophoretic analysis of VirD2 removal by HsTDP2. VirD2-ssDNA complexes were incubated without HsTDP2 or with increasing concentrations of HsTDP2, in the presence or absence of MgCl_2_. The reaction mixtures were resolved by SDS-PAGE on a 10% gel, and free VirD2 and VirD2-ssDNA complexes were visualized by fluorescence imaging (Fluo., top panel) and Western blotting (Western, middle panel). Free VirD2, VirD2-ssDNA complexes, and free HsTDP2 are indicated by cyan, magenta, and red arrowheads, respectively. The sizes of ssDNA liberated by HsTDP2 were analyzed by electrophoresis on TBE-Urea 15% gels (TBE-Urea 15%, bottom panel). 16-nt and 22-nt ssDNA oligomers are indicated by green and yellow arrowheads, respectively.

### Removal of VirD2 from a synthetic T-complex by plant TDP2

Next, we investigated whether VirD2 can be cleaved from the synthetic T-complex by TDP2 of a natural Agrobacterium host plant. To this end, we selected TDP2 of *Arabidopsis thaliana* (AtTDP2), tagged it with T7 and 6xHis epitopes, expressed it in *E. coli,* and partially purified it on a nickel-agarose resin under non-denaturing conditions to preserve the enzymatic activity. We then assayed the biochemical activity of AtTDP2. As a negative control, we utilized the AtTDP2 mutant H431N with the essential His-431 replaced with an asparagine residue; this conserved residue is required for the activity of mammalian TDP2 [28,36]. Coomassie blue staining of the AtTDP2 and H431N preparations before and after purification revealed similar electrophoretic patterns (Fig 4A), and their Western blot analysis using anti-T7 antibody indicated similar amounts of AtTDP2 in the wild-type and mutant samples to be used in the activity assays (Fig 4B).

**Fig 4:**
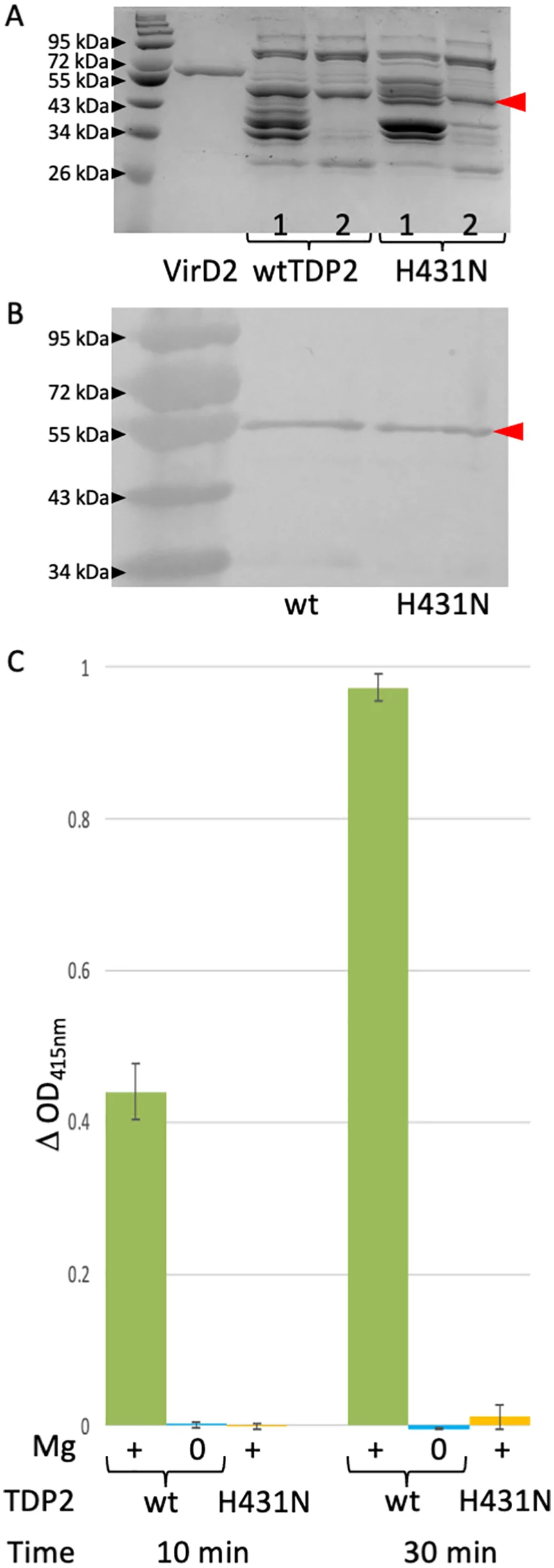
Partial purification and enzymatic activity of AtTDP2. **(A)** Electrophoretic analysis of purified VirD2 and partially purified AtTDP2 and its H431N mutant. The protein preparations were resolved by SDS-PAGE on a 10% gel, and visualized by Coomassie blue staining. Lanes 1 and 2, soluble proteins before and after purification, respectively. **(B)** Western blot analysis of partially purified AtTDP2 and its H431N mutant. AtTDP2 is indicated by red arrowheads. **(C)** AtTDP2 enzymatic activity. For quantification of the chromogenic T5PNP substrate, the OD_415nm_ measurements were normalized by subtracting the control values obtained in the absence of AtTDP2. The measurements were made at the indicated reaction times in the presence or absence of MgCl_2_. The error bars represent the standard deviation of the means from triplicate experiments; wt, wild-type; H431N, His-431 to Asn substitution mutant of AtTDP2.

The TDP2 enzymatic activity was measured using the chromogenic substrate thymidine 5′-monophosphate p-nitrophenyl (T5PNP) by a classic assay of phosphodiesterase activity [28,37,38], in which cleavage of the phosphodiester bond releases thymidine 5’-monophosphate and p-nitrophenol, and the latter is detected and quantified by spectrophotometry. Fig 4C shows that significant phosphodiesterase activity was measured with wild-type AtTDP2, and that this activity was reaction time-dependent and observed only in the presence of MgCl_2_. Almost no enzymatic activity was measured with H431N, suggesting that there was no other phosphodiesterase activity in the protein preparations.

Finally, for better characterization of AtTDP2, we investigated its subcellular localization in plant tissues (Fig 5). Consistent with sequence-based prediction using DeepLoc 2.0 (https://services.healthtech.dtu.dk/services/DeepLoc-2.0/) and the TDP2 known function in genomic DNA repair, transient expression of GFP-tagged AtTDP2 in *Nicotiana benthamiana* leaves showed strong nuclear accumulation of AtTDP2, with the fluorescent signal colocalizing with mRFP-tagged nuclear marker VirD2NLS but not with the cytoplasmic marker glutathione reductase (GSR) (Fig 5).

**Fig 5:**
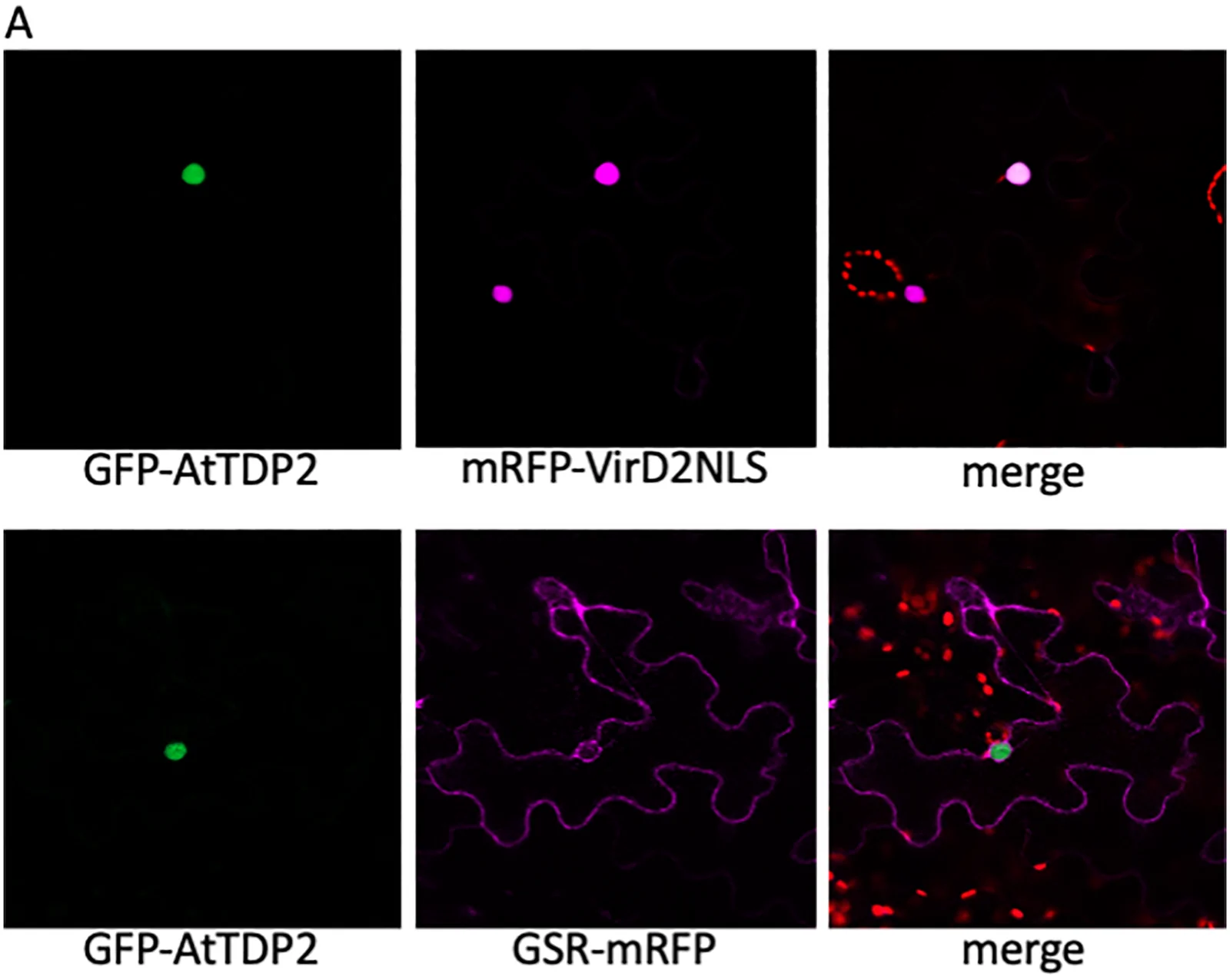
Subcellular localization of AtTDP2. GFP-AtTDP2 was transiently co-expressed with nuclear and cytoplasmic reference proteins mRFP-VirD2NLS or GSR-mRFP, respectively, in *N. benthamiana* leaves. GFP signal is in green; mRFP signal is in pink; merged GFP and mRFP signals are in light pink, and chlorophyll autofluorescence is in red. All images are single confocal sections.

Following its biochemical characterization, we investigated the ability of AtTDP2 to resect VirD2 from the synthetic T-complex, using the experimental pipeline described for HsTDP2. First, we verified that AtTDP2A, VirD2, and VirD2-ssDNA complexes can be separated and concurrently detected using Western blotting with anti-T7 antibody (Fig 6A). Fluorescence imaging of VirD2-ssDNA complexes incubated with increasing concentrations of wild-type AtTDP2 in the presence of MgCl_2_ showed a decrease in the amounts of the T-complex (Fig 6B, top panel). Similar disassembly of the T-complexes by AtTDP2 and a concurrent increase in the amount of free VirD2 was observed using Western blot analysis (Fig 6B, middle panel). This AtTDP2 activity was specific, as it absolutely depended on the presence of magnesium ions and was not observed with the H431N mutant of AtTDP2 (Fig 6B). Fluorescence imaging also revealed the presence of similar amounts of the unreacted 22-nt ssDNA substrate in all lanes, whereas the 16-nt fragment liberated following VirD2 removal from the T-complex was observed only in the presence of AtTDP2 and MgCl_2_ (Fig 6B, bottom panel). Similarly to experiments with HsTDP2, no shorter fluorescently labeled ssDNA species were observed, indicating that AtTDP2 specifically cleaved the phosphodiester bond between VirD2 Tyr-29 and the first nucleotide of the 16-nt ssDNA substrate molecule attached to VirD2, and that no other phosphodiesterases that can cleave non-specifically within the ssDNA substrate molecule were present in the protein preparations (Fig 6B, bottom panel). Taken together, these observations lend strong support to the idea that, during genetic transformation, plant TDP2 can remove VirD2 from the T-strand before its integration into the plant genome.

**Fig 6:**
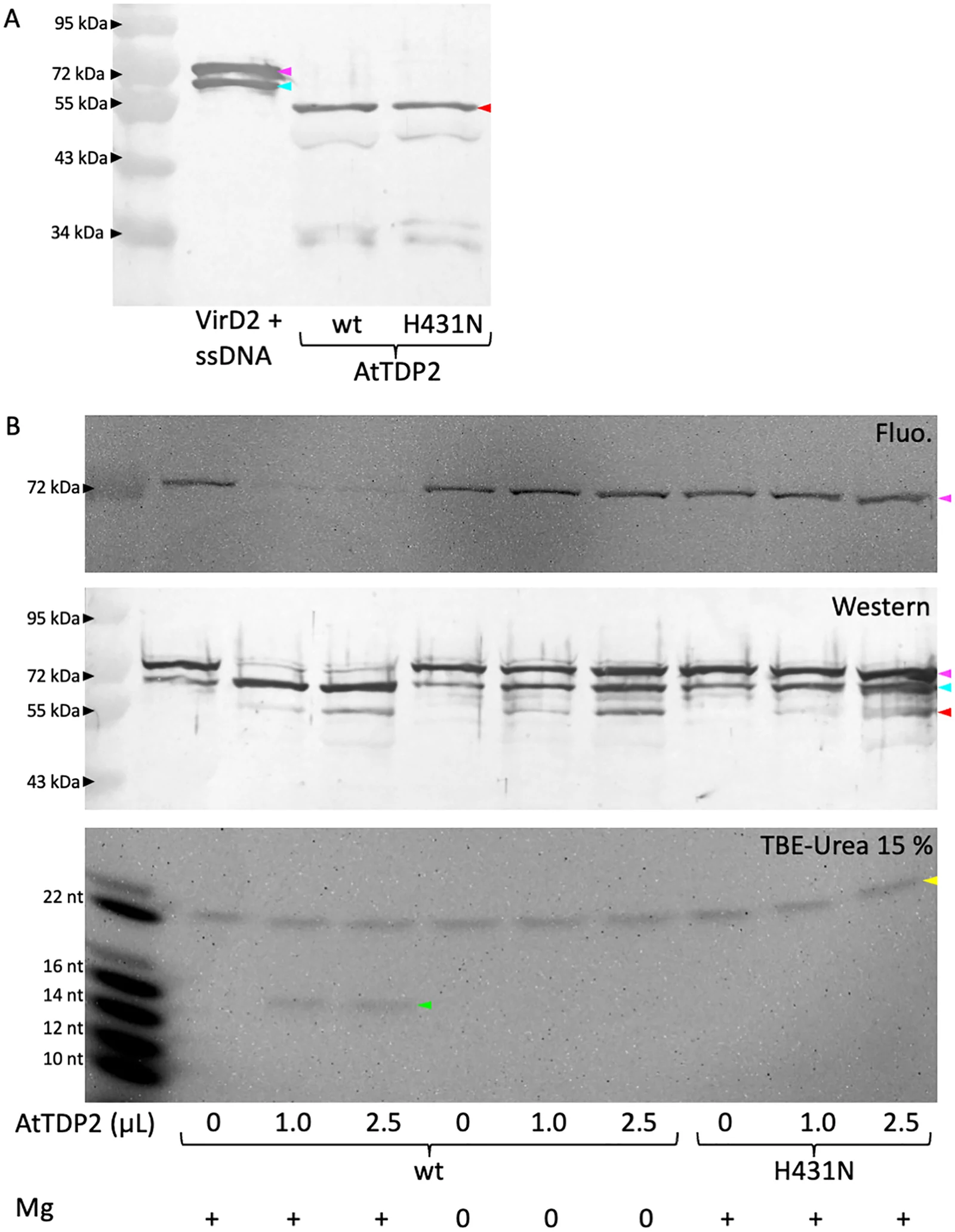
Removal of VirD2 from the synthetic T-complex by AtTDP2. **(A)** Western blot detection of VirD2-ssDNA, AtTDP2, and H431N using anti-T7-tag antibodies. **(B)** Electrophoretic analysis of VirD2 removal by AtTDP2. VirD2-ssDNA complexes were incubated without AtTDP2 or with increasing concentrations of AtTDP2, in the presence or absence of MgCl_2_. The reaction mixtures were resolved by SDS-PAGE on a 10% gel, and free VirD2 and VirD2-ssDNA complexes were visualized by fluorescence imaging (Fluo., top panel) and Western blotting (Western., middle panel). Free VirD2, VirD2-ssDNA complexes, and free AtTDP2 are indicated by cyan, magenta, and red arrowheads, respectively. The sizes of ssDNA liberated by AtTDP2 were analyzed by electrophoresis on TBE-Urea 15% gels (TBE-Urea 15%, bottom panel). 16-nt and 22-nt ssDNA oligomers are indicated by green and yellow arrowheads, respectively. wt, wild-type; H431N, His-431 to Asn substitution mutant of AtTDP2.

## Discussion

Direct assessment of the TDP2 ability to remove VirD2 from the T-complex requires development of an experimental technique for production of these complexes *in vitro*. *Agrobacterium* VirD2 is closely related to conjugative relaxases [34,39], a family of site-specific endonucleases involved in strand transfer during the process of conjugative DNA transfer [40]. As a site-specific endonuclease, VirD2 cleaves T-DNA borders between the 3^rd^ and the 4^th^ nt, and the downstream ssDNA segment, the T-strand, remains covalently attached at its 5’ end to the Tyrosine 29 residue of VirD2, Tyr-29 [23]. In *Agrobacterium* cells, VirD2 associates with VirD1 and likely with other proteins to liberate the T-strand from the Ti-plasmid [23,41]. However, *in vitro*, VirD2 can cleave ssDNA substrates containing the T-DNA border sequence without any cofactors, generating a VirD2-ssDNA complex [30–32]. Here, we built on previously published protocols, in which VirD2 is incubated with a 35-nt-long ssDNA radioactively labeled at its 3’-end for 1–3 h at 37°C [30–32], to develop a simple and flexible technique for production of fluorescently labeled synthetic T-complexes of variable lengths that are tractable and amenable to biochemical manipulation and simultaneous detection of the complexes and their individual protein and ssDNA components. Using this technique, we directly demonstrated that the Tyr-29 residue of VirD2 is required for its endonuclease activity, previously shown only by a DNA nicking activity in *E. coli* [35].

A typical T-DNA border is composed of 25 nucleotides and contains two highly conserved segments [42]. Using TraI, a relaxase closely related to VirD2, and a series of point mutations in the TraI 22-nt target sequence substrate, a 6-nt ATCCTG sequence in the second conserved region is crucial for TraI activity [43]. We used ssDNA substrates of various lengths to identify the minimal sequence required for the VirD2 activity. We found that an 8-nt ATCCTGTC oligomer, containing the 6-nt core and two nucleotides adjacent to the cleavage site that remain bound to VirD2, represented the ssDNA sequence necessary and sufficient for recognition and processing by VirD2. In addition, we observed that a shorter sequence upstream of the cleavage site resulted in higher efficiency of the reaction. This could happen for two reasons: either the reverse reaction is less efficient, or the cleavage reaction itself is more efficient with shorter nucleotide substrates (potentially because longer sequences may lead to secondary structures or because the VirD2 molecule may have to move along the DNA strand to position itself at the exact cleavage site). Besides the identification of these important parameters of the VirD2-mediated ssDNA cleavage and VirD2 covalent attachment to ssDNA, we used the synthetic T-complex to better understand how the 5’ end of its ssDNA is liberated from the attached VirD2 for subsequent integration.

TDP2, encoding the tyrosyl-DNA phosphodiesterase 2 activity, was first identified in mammalian systems as an enzyme involved in DNA repair, particularly in removing topoisomerase II (TOP2)-induced DNA adducts [27]. TOP2 creates temporary DNA breaks to relieve torsional stress during DNA replication and transcription and can bind covalently to DNA [44], forming a DNA-protein crosslink. TDP2 can resolve these TOP2-DNA adducts by specifically cleaving the phosphodiester bond between a nucleotide and a tyrosine residue of TOP2; the DNA break can then be repaired via the classical NHEJ pathway, thus preventing genomic instability [44]. TDP2 can act on a variety of substrates such as single- or double-stranded DNA [36,45]; structural studies suggest that TDP2 can bind ssDNA and requires at least two unpaired nucleotides at the 5’-end of a 5’-tyrosine adduct [46]. TDP2 also likely plays a role in the life cycle of picornaviruses, where it removes the viral VPg protein from the 5’-end of the viral genomic RNA [47,48]. Whereas TDP2 has been mostly studied in mammalian systems, TDP2 is widely conserved in the animal and plant kingdoms (Fig S2), and, similarly to mammalian TDP2, *Arabidopsis* TDP2 is involved in the repair of TOP2-DNA crosslinks [29]. Based on this activity, plant TDP2 was suggested to play a role during *Agrobacterium* T-DNA integration, representing one of the pathways for removing VirD2 from the T-complex [24]. Although TDP2 is not essential for T-DNA integration, *Agrobacterium*-mediated genetic transformation is severely impaired in *mre11*/*tdp2* double mutant plants, in which both the TDP2 and MRN pathways are disabled. In a TDP2 single mutant, the spectrum of RB-genome junctions was shifted toward a typical TMEJ profile [24]. Whereas genetic evidence has implicated TDP2 in T-DNA integration [24], our in vitro experiments demonstrate the TDP2 enzymatic activity in removing VirD2 from the *Agrobacterium* T-complex, thus providing important biochemical evidence of the TDP2 role in T-DNA integration.

We showed that the enzymatic activity of TDP2 from *Arabidopsis* requires the presence of magnesium ions, consistent with the known requirement for divalent cations for the activity of mammalian TDP2 [36]. Another requirement for the AtTDP2 function is its His-431 residue, and an H431N substitution mutation abolished the protein phosphodiesterase activity. The His-431residue of AtTDP2 corresponds to a highly conserved position in TDP2 proteins, such as His-351 in HsTDP2, and its mutation disables TDP2 activity in mammalian systems [28,36]. Both HsTDP2 and AtTDP2 were able to cleave VirD2-ssDNA complexes, severing VirD2 from the attached ssDNA. Analyses of the sizes of ssDNA molecules released from the T-complex by TDP2 activity demonstrated that TDP2 hydrolyzes specifically the phosphodiester bond between Tyr-29 of VirD2 and the nucleotide at the 5’-end of the attached ssDNA and does not display non-specific endonuclease activity. Although it has been shown that VirD2 is not sufficient to mediate T-DNA integration by itself but requires the action of plant factors [49], it is likely involved in the T-DNA integration process. Early studies have shown that several VirD2 mutants could affect the integration process [32,50]; it would be interesting in future works to assess if TDP2 activity is modified with complexes generated with VirD2 harboring these mutations. That both human and plant TDP2s could process the VirD2-ssDNA complex shows that this activity is evolutionarily conserved between animals and plants and is consistent with the ability of *Agrobacterium* to transfer and integrate T-DNA into cells of many eukaryotic species, including human cells [51].

Historically, TDP2 activity *in vitro* was measured mostly with phosphotyrosyl-ssDNA substrates [28,37,38], and only one study employed protein-DNA adducts purified from yeast cells [45]. To our knowledge, it is the first time that TDP2 activity is observed using a synthetic protein-ssDNA complex as substrate in general, and the *Agrobacterium* synthetic T-complex, in particular. Because these complexes are more realistic than phosphotyrosyl-ssDNA conjugates, their easy generation with ssDNA of different lengths and compositions, which can be attached to a marker of choice, will likely be useful for studies of plant as well as animal TDP2 enzymes. Using this system, we showed that AtTDP2 can specifically sever VirD2 from T-complexes. Whereas most recent approaches to studies of *Agrobacterium*-mediated genetic transformation employ genetic and cell biological techniques to investigate transient and stable T-DNA expression, bacterial-host protein interactions, and T-DNA integration, biochemical *in vitro* studies using defined molecular components are crucial to explore the molecular mechanisms involved in the transformation process.

## Materials and methods

### Plasmid construction

For protein expression in *E. coli*, the full-length *vir*D2 coding sequence from *Agrobacterium tumefaciens* strain C58 (C58*vir*D2, Atu6182) was PCR-amplified from a purified C58 Ti-plasmid, digested with EcoRI-XhoI, and cloned into the same sites of pET28a(+) (Clontech), resulting in pET28-C58*vir*D2. The construct expressing C58-VirD2 carrying a Y29A mutation (pET28-C58*vir*D2Y29A) was obtained by whole-plasmid PCR amplification using pET28-C58*vir*D2 as template, with two primers that reverse-complement each other, introducing the mutation. The full-length TDP2 sequence of *Arabidopsis thaliana* (AtTDP2, At1g11800) was PCR-amplified from a cDNA library, digested with EcoRI-SalI, and introduced into the EcoR1-Xho1 sites of pET28a(+), generating pET28-AtTDP2. The construct expressing AtTDP2 carrying an H431N mutation was obtained by whole-plasmid PCR amplification with reverse-complementing primers, introducing the mutation and resulting in pET28-AtTDP2H431N. For transient expression of GFP-tagged proteins in plants, AtTDP2 was inserted into the EcoR1-BamH1 sites of the binary plasmid pCB302T-GFP [52], resulting in pCB302T-GFP-AtTDP2. For this last construct, In-fusion SNAP assembly (Takara ST2320) was used following the manufacturer’s instructions. All PCRs were performed using the Ultra-Pfu II polymerase (Agilent Technologies 600672–51), and construct sequences were verified by DNA sequencing. All primer sequences are listed in Table S2. The plasmids pRCS2-ocs-bar-GSR-mRFP and pRCS2-ocs-bar-mRFP-VirD2NLS, for plant transient expression of cytoplasmic marker GSR-mRFP and nuclear marker mRFP-VirD2NLS, respectively, were obtained from Dr. Stanton B. Gelvin (Purdue University).

### Recombinant histidine-tagged protein purification

All proteins were expressed in the *E. coli* strain T7-express (NEB C2566) transformed with the pET28-derived constructs described above. For C58-VirD2 and C58-VirD2Y29A, 2 mL of an overnight seed culture were added to 50 mL of 2xYT medium supplemented with 50 mg.L^-1^ kanamycin, grown for 1–2 h at 37°C, 240 rpm until an OD_600nm_=0.4-0.6, expression was induced by adding IPTG (0.5 mM final concentration), and the culture was grown for another 6 h at 28°C, 240 rpm before harvesting the cells by centrifugation. The pellet was resuspended in lysis buffer (NEB P8116) supplemented with 1 mM PMSF and 1 mM ß-mercaptoethanol, 6 µL T4-lysozyme (NEB P8115) was added, the suspension was incubated for 45 min at 4°C on a rotary shaker, then 2 µL Benzonase nuclease (Millipore/Novagen 70746–3) was added, and the incubation was continued for 15 min at 4°C. After centrifugation (10 min, 16000xg, 4 °C), the supernatant was discarded and the pellet resuspended in 4 mL of 50 mM Tris-HCl pH 8, 1 M NaCl, 1 mM PMSF, and 1 mM ß-mercaptoethanol, centrifuged again, and the pellet was resuspended in 4 mL of 50 mM Tris-HCl pH 8, 1 M NaCl, 4 M urea, 1 mM PMSF and 1 mM ß-mercaptoethanol. After centrifugation (10 min, 16000xg, 4 °C), the supernatant was recovered, imidazole (5 mM final concentration) and 1 mL of nickel-agarose resin (Qiagen 30210) were added, and the solution was incubated for 1 h at 4°C on a rotary shaker. The mixture was transferred to a column (Biorad, 7311550), the resin and the adsorbed protein were allowed to settle down, washed with 50 mL of 50 mM Tris-HCl pH 8, 1 M NaCl, 4 M urea, 10 mM imidazole, 1 mM PMSF, and 1 mM ß-mercaptoethanol, and the protein was eluted in 2 mL of the same buffer containing 250 mM imidazole. The eluate was then dialyzed using a D-tube dialyzer, maxi MWCO 12–14 kDa (Millipore 71510–3), for 20 h at 4°C in 50 mM Tris-HCl pH 8, 100 mM NaCl, 10% glycerol, 1 mM PMSF, and 1 mM ß-mercaptoethanol. For AtTDP2 and AtTDP2H431N, 2 mL of an overnight seed culture were added to 50 mL of 2xYT medium, supplemented with 50 mg.L^-1^ kanamycin, and grown for 1–2 h at 37°C, 240 rpm until an OD_600nm_=0.4-0.6; protein expression was induced by adding IPTG (0.5 mM final concentration), and the culture was grown for 6 h at 28°C, 240 rpm before harvesting the cells by centrifugation. The pellet was resuspended in the lysis buffer (NEB P8116) supplemented with 1 mM PMSF and 1 mM ß-mercaptoethanol; 6 µL T4-lysozyme was added, and the suspension was incubated for 30 min at 4°C on a rotary shaker; then 2 µL Benzonase nuclease (Millipore/Novagen 70746–3) was added, and the incubation continued for 15 min at 4°C. After centrifugation (10 min, 16000xg, 4°C), the supernatant was recovered; imidazole (10 mM final concentration) was added, and the solution was mixed with 1 mL of nickel-agarose resin previously equilibrated in the lysis buffer, and incubated for 1 h at 4°C on a rotary shaker. The mixture was then transferred to a column, the resin and adsorbed protein were allowed to settle down, and washed with 50 mL of 50 mM Tris-HCl pH 8, 100 mM NaCl, 10 mM imidazole, 1 mM PMSF, and 1 mM ß-mercaptoethanol, and the protein was eluted in 2 mL of the same buffer containing 10% glycerol and 250 mM imidazole. The eluate was concentrated using an Amicon Ultra-4 centrifugal filter 50 KDa (Millipore UFC805024) following the manufacturer’s instructions. After purification, all protein solutions were aliquoted, snap-frozen in liquid nitrogen, and stored at -80°C. Protein concentrations were measured by the bicinchoninic acid (BCA) assay, using Pierce BCA Protein Assay Kit (Pierce, 23227) according to the manufacturer’s instructions.

### Generation of synthetic T-complexes

Approximately 0.5 nmol (27 µg) of purified VirD2 was mixed with 0.5 nmol of ssDNA oligomers, in a total volume of 500 µL of reaction buffer (20 mM Tris-HCl pH 8.5, 50 mM NaCl, 5 mM MgCl_2_, 1 mM DTT), at 25°C or 37°C for 1 or 3 h. Oligomers of different sizes and sequences listed in S1 Table and labeled with a FAM (5’-Fluorescein Phosphoramidite) group at their 3’-end were procured from Integrated DNA Technologies (IDT). The reaction was then stopped by placing the samples on ice and concentrated by centrifugation (15 min, 4°C, 14,000 RCF) using centrifugal filter units Amicon Ultra 0.5 mL (Millipore) with a 50 kDa nominal molecular weight limit. The concentrated samples (20 µL) were recovered by centrifugation of the column in an inverted position (4 min, 4°C, 1,000 RCF) and either mixed with Laemmli buffer and stored at -20°C for later analyses or used immediately for the TDP2 activity assays.

### Tyrosyl-phosphodiesterase activity assay

The tyrosyl-phosphodiesterase activity of partially purified *Arabidopsis* TDP2 was assayed using the chromogenic substrate T5PNP, thymidine 5′-monophosphate p-nitrophenyl (Sigma T4510). 0.5 µL of TDP2-enriched protein extract (0.75 µg total protein) was added to 40 µL of reaction buffer (50 mM Tris-HCl pH 7.5, 1 mM MgCl_2_, 50 mM KCl, 0.1 mg.mL^-1^ BSA, and 1 mM DTT) containing 5 mM of T5PNP; MgCl_2_ was omitted, and 1 mM EDTA was added as indicated in relevant experiments. The reaction mixture was incubated for 10 or 30 min at RT, and the reaction was stopped by adding 100 µL of 1 M Na_2_CO_3_ and placing the mixture at 4°C. The nitrophenyl product concentration was determined by measuring OD_415nm_, using the reaction mixture without the TDP2 extract as blank. All measurements were performed in triplicate.

### Assay of VirD2-ssDNA cleavage by TDP2

5 µL of concentrated VirD2-ssDNA complex preparation was incubated either alone or with 1.0, 2.5, or 5.0 µL of either commercial human TDP2 (Sigma SRP0438, His-GST tagged) or partially purified *Arabidopsis* TDP2 (total protein concentration 1.5 µg.µL^-1^) for 45 min at 25°C in 40 µL of reaction buffer (50 mM Tris-HCl pH 7.5, 1 mM MgCl_2_, 50 mM KCl, 0.1 mg.mL^-1^ BSA, 1 mM DTT). The reaction was stopped by placing the samples at 4°C, and samples were divided into 10 µL aliquots, mixed with 10 µL of either Laemmli sample buffer or TBE-Urea sample buffer (Invitrogen LC6876), and stored at -20°C before electrophoretic analysis.

### Visualization of proteins and protein-ssDNA complexes

Samples resuspended in Laemmli buffer were heated for 5 min at 100°C and analyzed by SDS-PAGE on a 10% acrylamide gel (80V, 2–3 h at room temperature), protected from light. Fluorescence was detected using a Bio-Rad gel visualizer (GelDoc XR+) with an Xcitablue converter screen (Biorad 1708081) and SYBR Green settings (excitation peak 440 nm, emission filter 535–640 nm). The same gel was then either stained with Coomassie blue for protein detection or analyzed by Western blotting. For Western blots, proteins were transferred onto a nitrocellulose membrane (Amersham Protran premium, 0.45 µM NC, 10600003) and incubated for 16 h at 4°C with either anti-T7-tag polyclonal antibody (Invitrogen 32386, 1/10000 titer) or anti-6xHis polyclonal antibody (Genscript A00174, 1/1000 titer). Incubation with the secondary antibody, HRP (horseradish peroxidase)-conjugated goat-anti-rabbit antibody (Invitrogen 31460, 1/2500 titer), was performed for 1 h at 25°C. HRP activity was detected directly on the membrane using a 1-step ultra TMB-blotting solution (Thermo Scientific 37574) following the manufacturer’s instructions.

### Visualization of ssDNA oligomers

Samples resuspended in TBE-Urea sample buffer were heated for 3 min at 70°C and resolved by electrophoresis on a 15% polyacrylamide TBE-Urea gel (Invitrogen EC6885), protected from light, for 1 h at 180V in TBE buffer. The size marker mixture was prepared using five fluorescently labeled 22-nt, 16-nt, 14-nt, 12-nt, and 10-nt ssDNA oligomers (1 mM each) with sequences listed in S1 Table. After electrophoresis, fluorescence was detected on the gel as above, using a Bio-Rad gel visualizer (GelDoc XR+) with an Xcitablue converter screen and SYBR Green settings.

### Transient expression in plant tissues and confocal microscopy

*A. tumefaciens* strain EHA105 transformed with binary plasmids expressing GFP- or mRFP-tagged proteins was grown overnight at 28°C, centrifuged, and the pellet resuspended in MES buffer (10 mM MgCl_2_, 10 mM MES, pH 5.6). After 2 h incubation at 25°C, the cell concentration was adjusted to OD_600nm_ = 0.25 and infiltrated into intact leaves of 4–6-week-old soil-grown *Nicotiana benthamiana* plants. Three days after agroinfiltration, the infiltrated areas of the leaves were harvested and analyzed under a Zeiss LSM900 Pascal laser scanning confocal microscope equipped with a super-resolution Airyscan 2 detection system. The excitation wavelengths for GFP/plastid autofluorescence and mRFP were 488 nm and 561 nm, respectively, and the detection wavelengths were 410–546 nm for GFP, 656–700 nm for autofluorescence, and 554–621 nm for mRFP; the pinhole was set at 1 AU (Airy unit) and detector gain at 700 V.

## Supporting information

S1 Fig Flowchart representing the experimental procedure for detection of TDP2-mediated removal of VirD2 from the synthetic T-complex. (**A**) Step 1: VirD2-ssDNA complex is synthesized as described in Fig 1A, with a 22-nt ssDNA substrate fluorescently labeled with FAM at its 3’-end, resulting in the formation of synthetic T-complexes comprised of VirD2 covalently attached to a 16-nt ssDNA segment. These complexes are concentrated by centrifugation using a Centricon device. A 6-nt unlabeled ssDNA segment is released after this reaction. (**B**) Step 2: Synthetic T-complexes are incubated with TDP2 in the TDP2 reaction buffer, resulting in free VirD2 and a fluorescently labeled 16-nt ssDNA segment, while the unreacted 22-nt ssDNA segment, the residual amounts of the synthetic T-complex, and the 6-nt ssDNA segment released after the reaction are still present in the reaction mixture. (**C**) Step 3: Samples are aliquoted in either Laemmli sample buffer for separation by SDS-PAGE on 10% polyacrylamide gels, followed by fluorescence imaging and western blot analysis, or TBE-Urea sample buffer for resolution by TBE-Urea 15% gel electrophoresis to detect small fluorescently labeled ssDNA segments, e.g., 22-nt and 16-nt ssDNA segments.
(PDF)

S2 Fig Alignment of AtTDP2 and HsTDP2 protein sequences. The alignment was performed using the MUSCLE software (https://www.ebi.ac.uk/jdispatcher/msa/muscle?stype=protein). The conserved essential histidine residue close to the protein C-terminus is highlighted in red. Asterisks indicate identical residues; colons indicate strongly conserved residues; periods indicate semi-conserved residues.
(PDF)

S1 Table Sequences of fluorescently labeled ssDNA segments used for experiments. The two conserved regions of RB T-DNA are indicated in green; the segment that remains attached to VirD2 is indicated in red.
(DOCX)

S2 Table Sequences of primers used for cloning. (DOCX)

S1 Data Original data for Fig 4C. (XLSX)

S2 Data Raw gels and blots. (PDF)
