# Supplementary material for: Removal of VirD2 from a synthetic T-DNA complex of Agrobacterium by the host DNA repair protein TDP2
Source: PLoS Pathog. 2026 Oct 26;22(10):e1014750. doi: 10.1371/journal.ppat.1014750 (PMC13727043; doi:10.1371/journal.ppat.1014750)
Supplement: Supplementary file 1 [file ppat.1014750.s001.pdf]

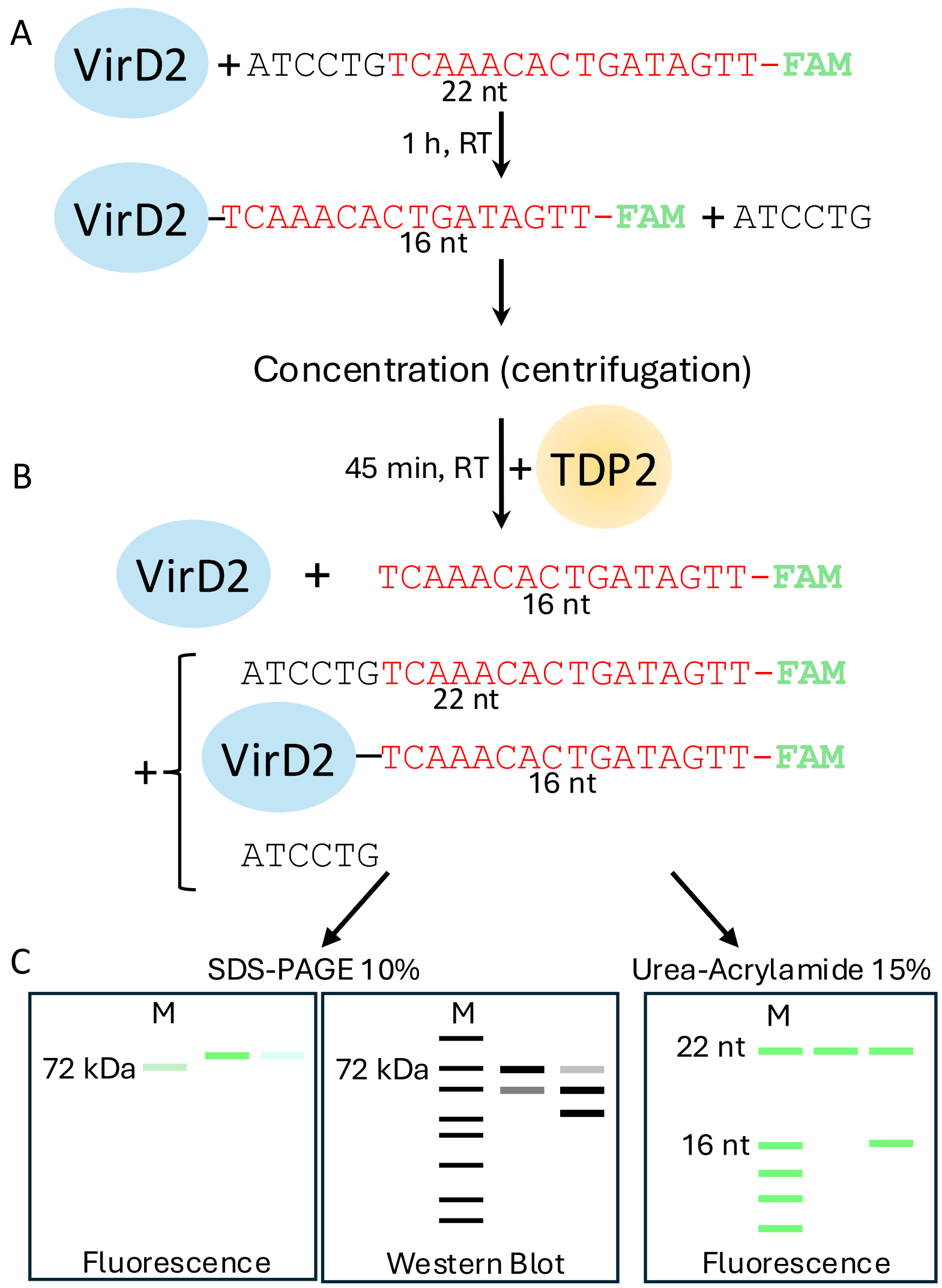

A
VirD2
+ ATCCTGTCAAACACTGATAGTT-FAM
22 nt
1 h, RT
VirD2 -TCAAACACTGATAGTT-FAM + ATCCTG
16 nt
Concentration (centrifugation)
B
45 min, RT
+ TDP2
VirD2 + TCAAACACTGATAGTT-FAM
16 nt
ATCCTGTCAAACACTGATAGTT-FAM
22 nt
+ VirD2 -TCAAACACTGATAGTT-FAM
16 nt
ATCCTG
C
SDS-PAGE 10%
Urea-Acrylamide 15%
M
72 kDa
Fluorescence
M
72 kDa
Western Blot
M
22 nt
16 nt
Fluorescence
