# Supplementary material for: Removal of VirD2 from a synthetic T-DNA complex of Agrobacterium by the host DNA repair protein TDP2
Source: PLoS Pathog. 2026 Oct 26;22(10):e1014750. doi: 10.1371/journal.ppat.1014750 (PMC13727043; doi:10.1371/journal.ppat.1014750)
Supplement: Supplementary file 2 [file ppat.1014750.s002.pdf]

```
At   MSTTIRFLRIVTSRAMSSSWSCNKCTFLNSASQKLN--CMICLAPVSLPSLSPPPPSLSI
Hs   -------------MELGSCLEGGREAAEEEGEPEVKKRRLLCVEFASVASCDAAVAQCFL
                    :.*. . .. :  :... :::   ::*:  .*:.* ... ..  :

At   SANDEAKWACKACTFLNTYKNSICDVCGTRSPTSSLLGFQDLTDSGLESNDADSSVGSVF
Hs   AEND---WEMERA--LNSYFEPPVE-----------------ESALERRPETISEPKTY
     : **    *  :  .  **:* :.  :                  :*.** .    *  ..:

At   FPLRRCIKRKAMDDDVVEVDGASVVCSESQGVMKKNKEIETKGVASDSGTPLTCLKILSY
Hs   VDL--------TNEETTDSTTSKISPSED--------------TQQENGS------MFSL
     . *         ::::::   :.:  **.              . .:.*:      ::* 

At   NVWFREDLELN---LRMRAIGHLIQLHSPHLICFQEVTPEIYDIFRKSNWWKAYSCSVSV
Hs   ITWNIDGLDLNNLSERARGVCSYLALYSPDVIFLQEVIPPYYSYLKKR------SSNYEI
      .*  :.*:**    * *.:   : *:** :* :*** *  *. :.*       *.. .:

At   DVAVSRGYYCMLL---SKLGVKSFSSKSFGNSIMGRELSIAEVEVPGRKPLVFATSHLES
Hs   ITGHEEGYFTAIMLKKSRVKLKSQEIIPFPSTKMMRNLLCVHVNVSGNE-LCLMTSHLES
      .. . **:  ::   *.: :** .  .* .: * *:*  . *:*.*.: * : ******

At   PCPGPPKWDQMFSRERVEQAKEAIEILR---PNANVIFGGDMNWCDKLDGKF-PLPDKWV
Hs   --------TRGHAAERMNQLKMVLKKMQEAPESATVIFAGDTNLRDREVTRCGGLPNNIV
              . .: **::* * .:: :.    .*.***.** *  *.   .   **:: *

At   DVWEVL-KPGDLGFTYDTKANPMLSGNRALQKRLDRILCRLDDYKLGGIEMVGKEAIPGL
Hs   DVWEFLGKPKHCQYTWDTQMNSNLGITAACKLRFDRIFFR-------------AAAEEGH
     ****.* **    :*:**: *. *. . * : *:***: *               *  * 

At   SYVKEKKVRGDIKKLELPVLPSDHFGLLVTLSPK-
Hs   IIPRSLDLLG-LEKLDCGRFPSDHWGLLCNLDIIL
        .. .: * ::**:   :****:*** .*.
```
