# Supplementary material for: Removal of VirD2 from a synthetic T-DNA complex of Agrobacterium by the host DNA repair protein TDP2
Source: PLoS Pathog. 2026 Oct 26;22(10):e1014750. doi: 10.1371/journal.ppat.1014750 (PMC13727043; doi:10.1371/journal.ppat.1014750)

Fig. 1B

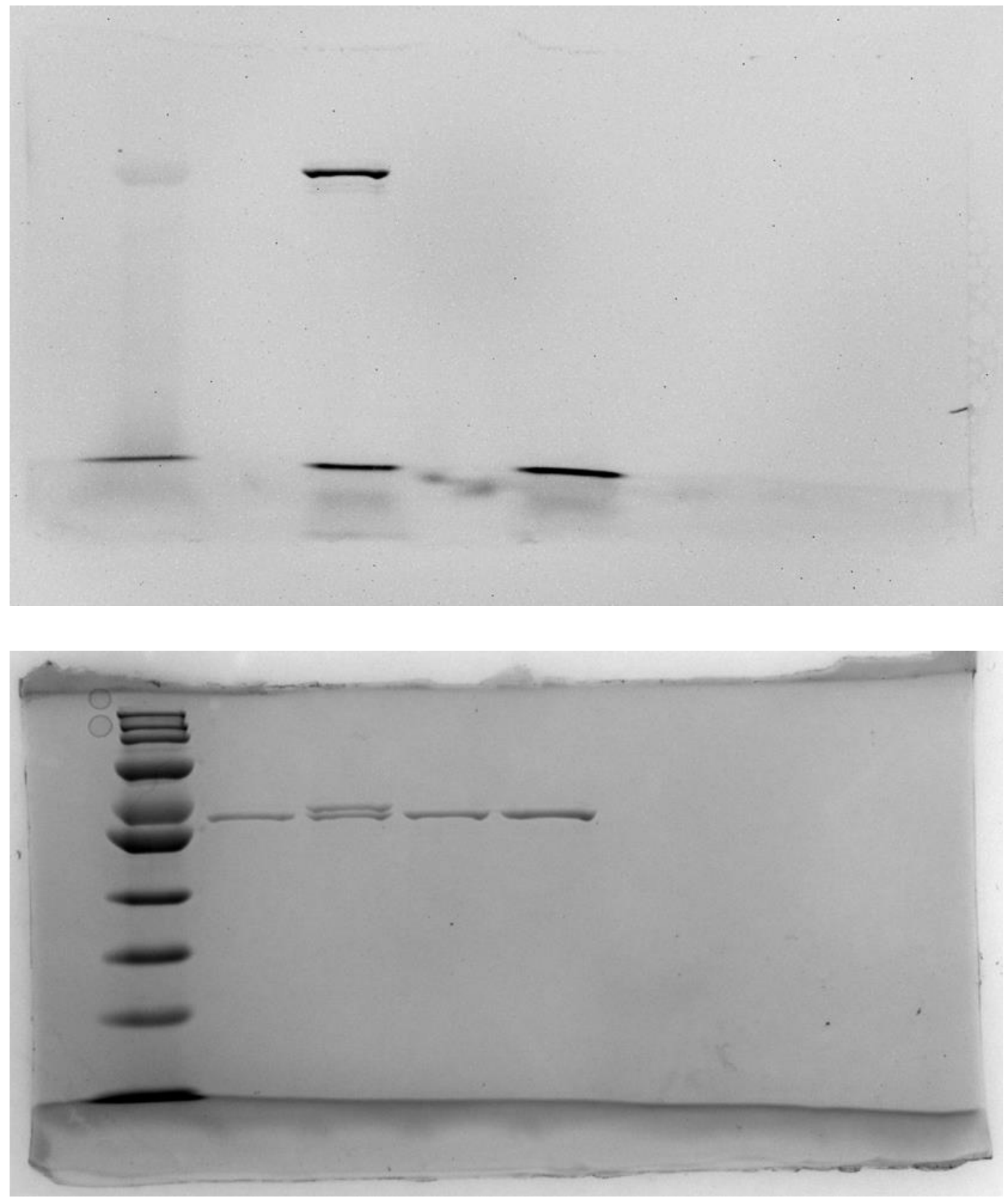

Fig. 1C

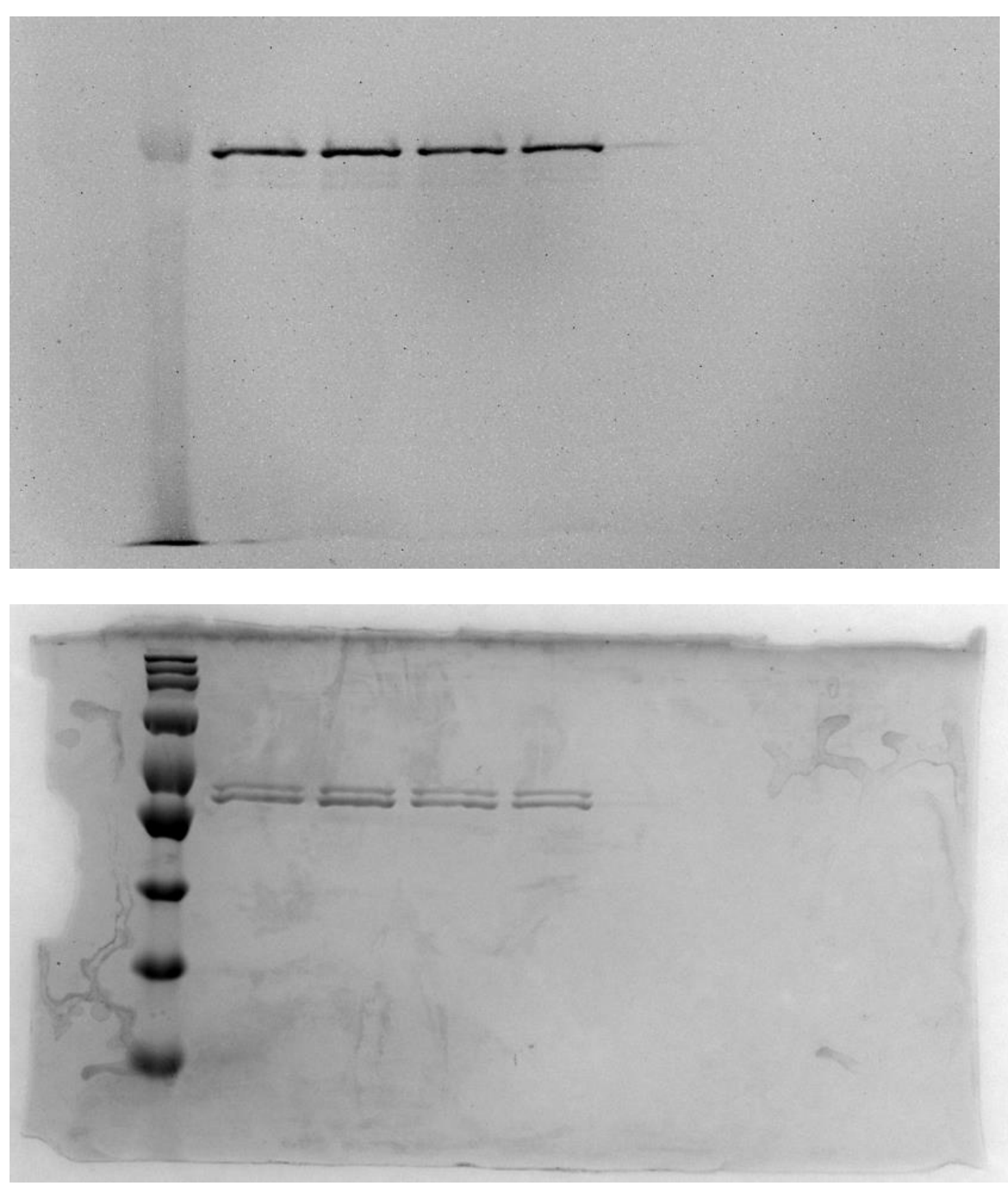

Fig. 2 A

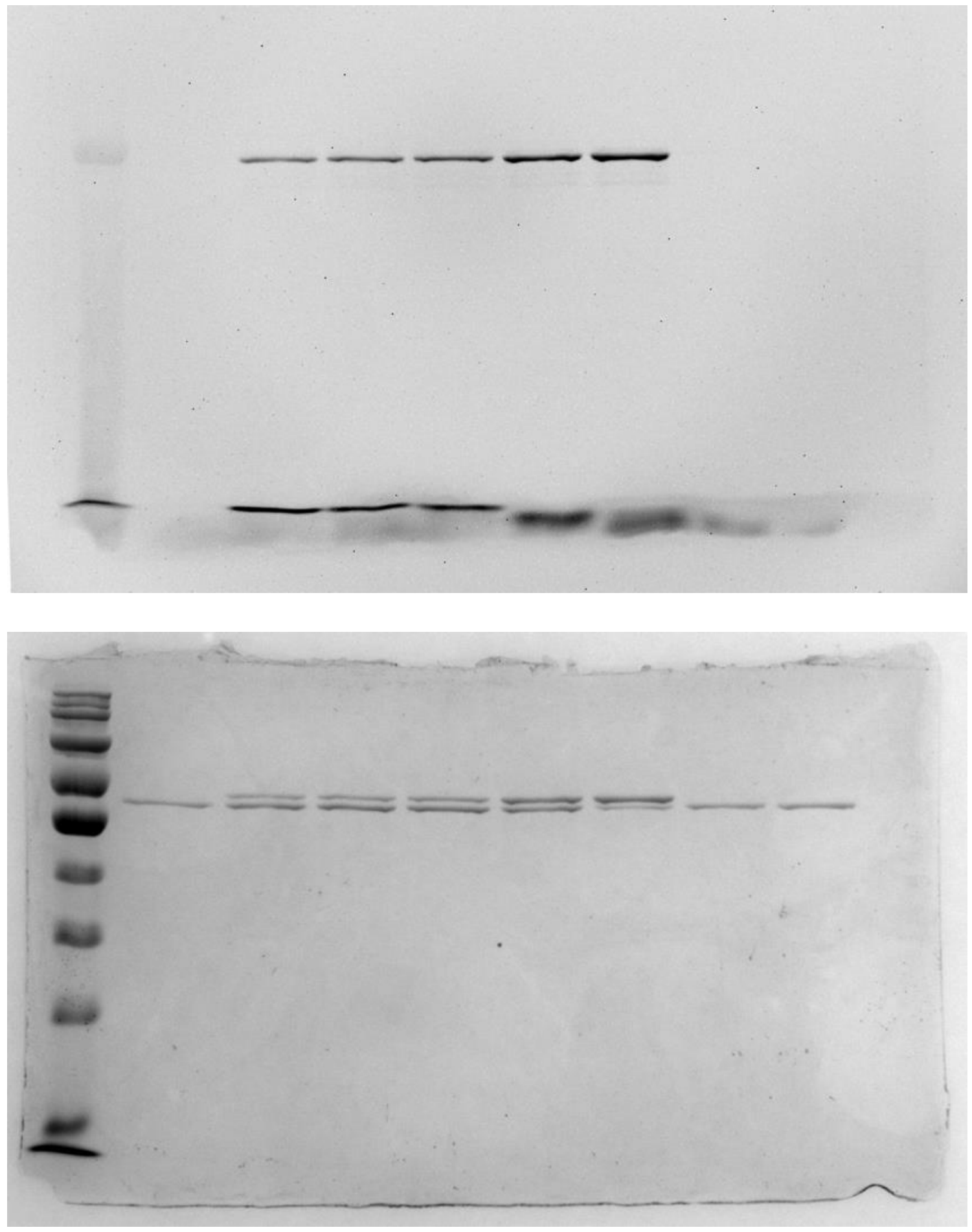

Fig. 2 B

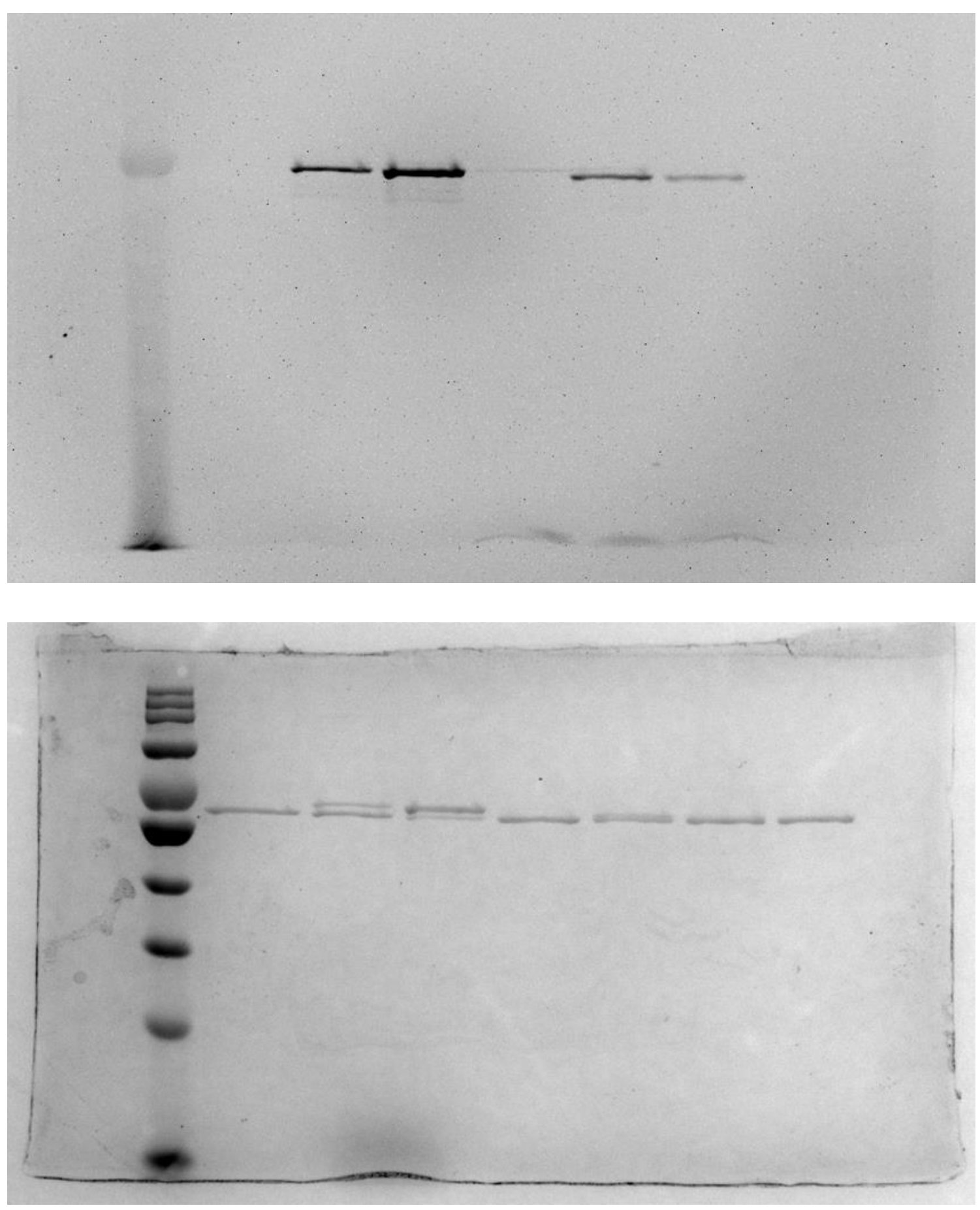

Fig. 2 C

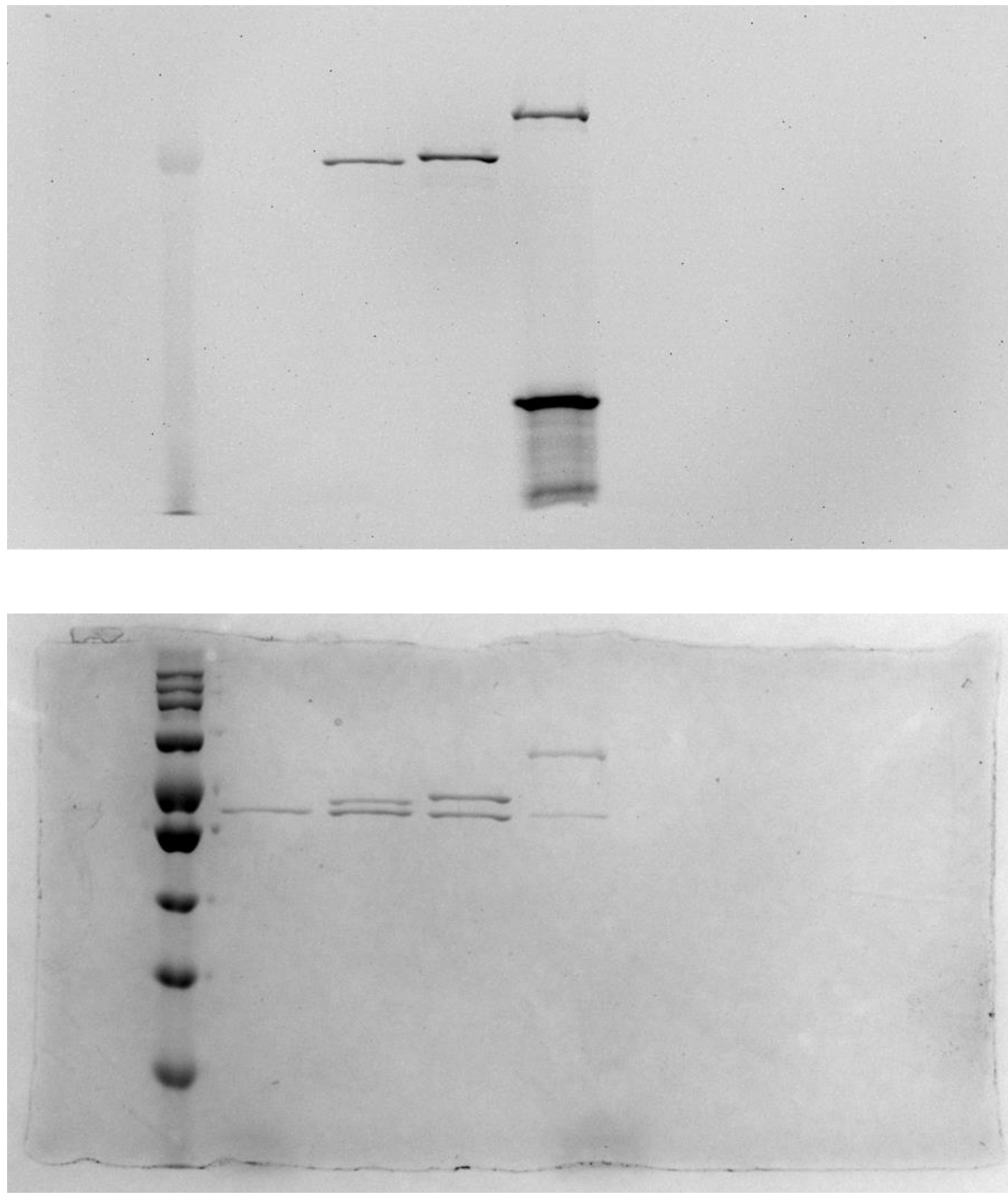

Fig. 3A

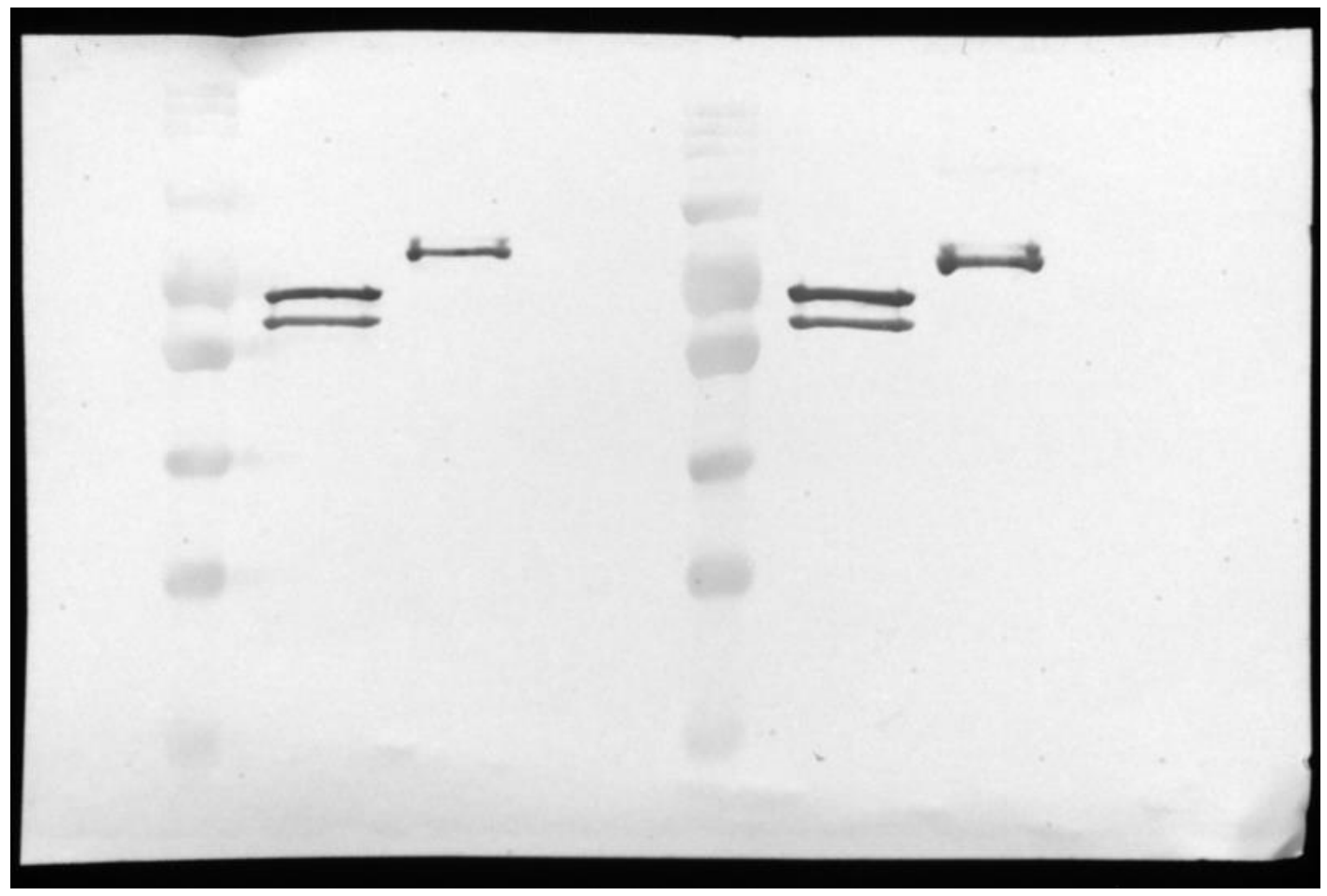

Fig. 3B

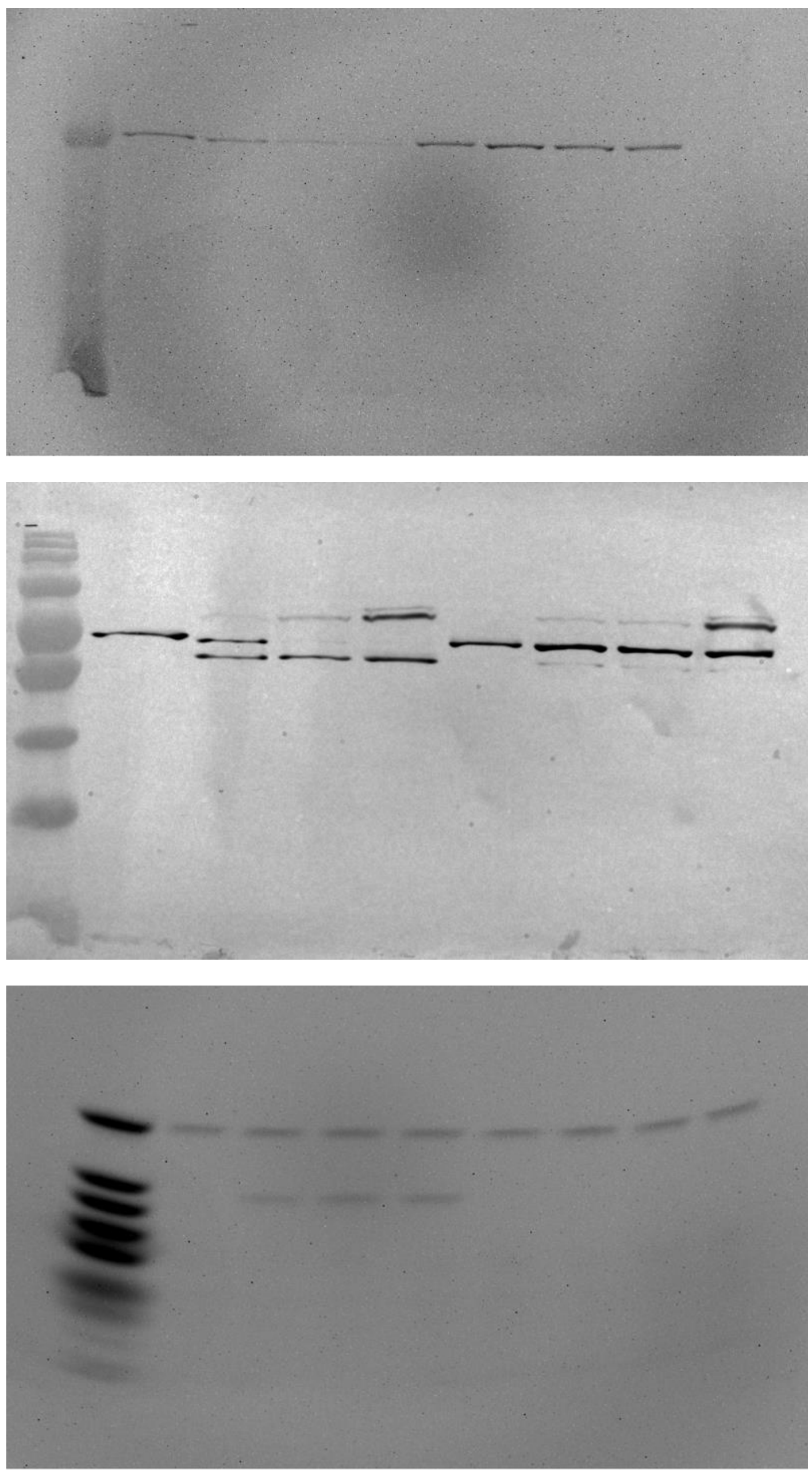

Fig. 4A

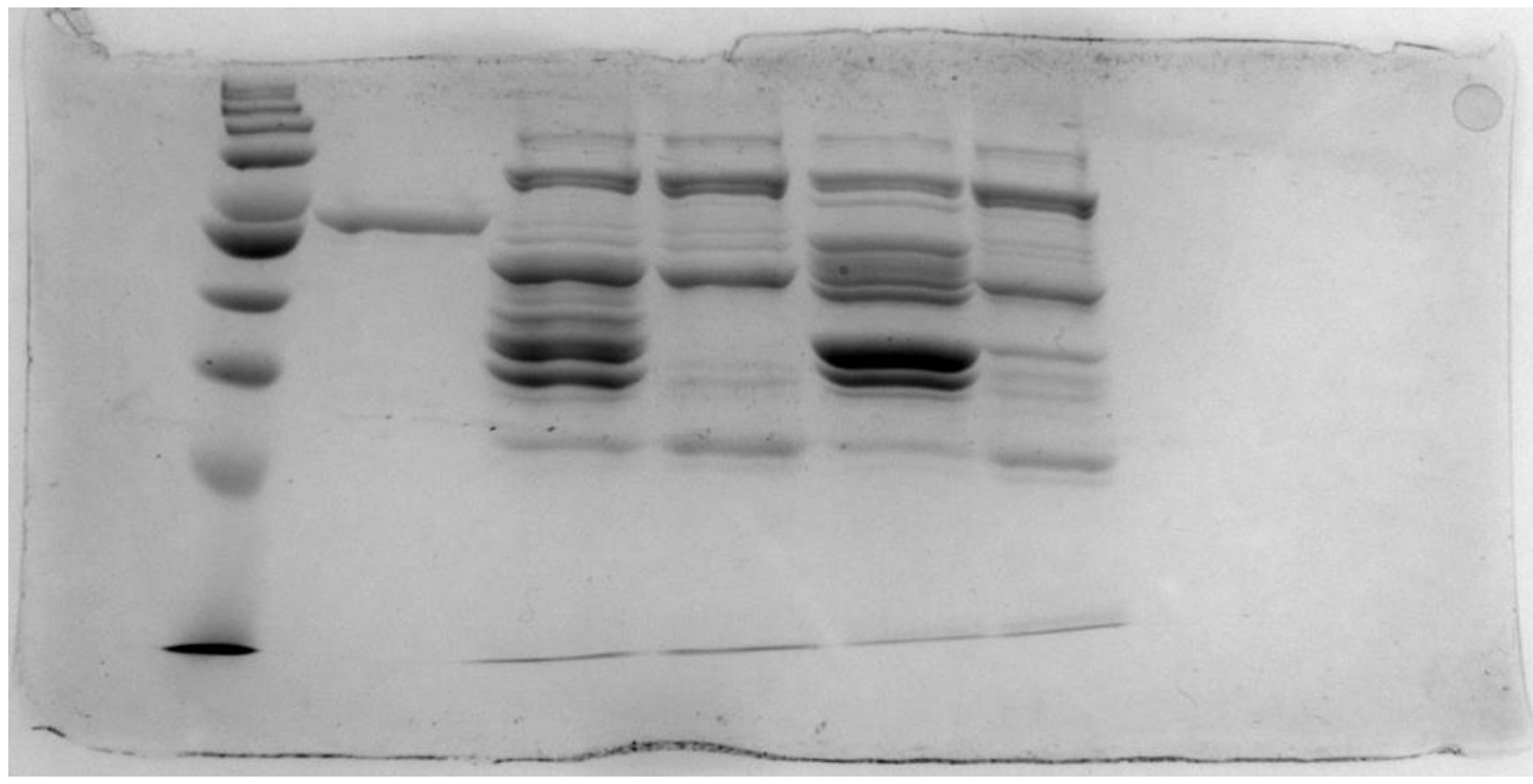

Fig. 4B

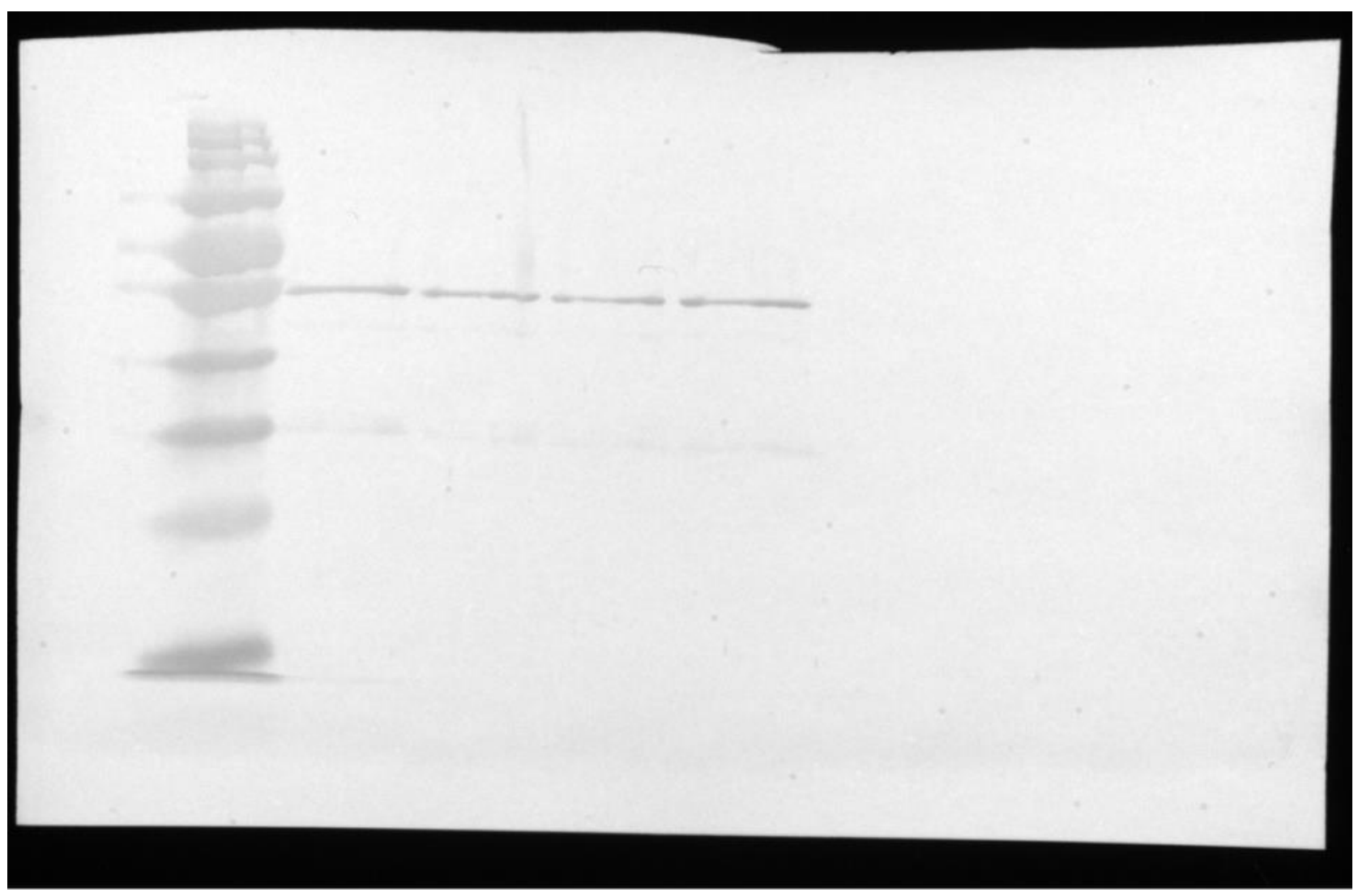

Fig. 6A

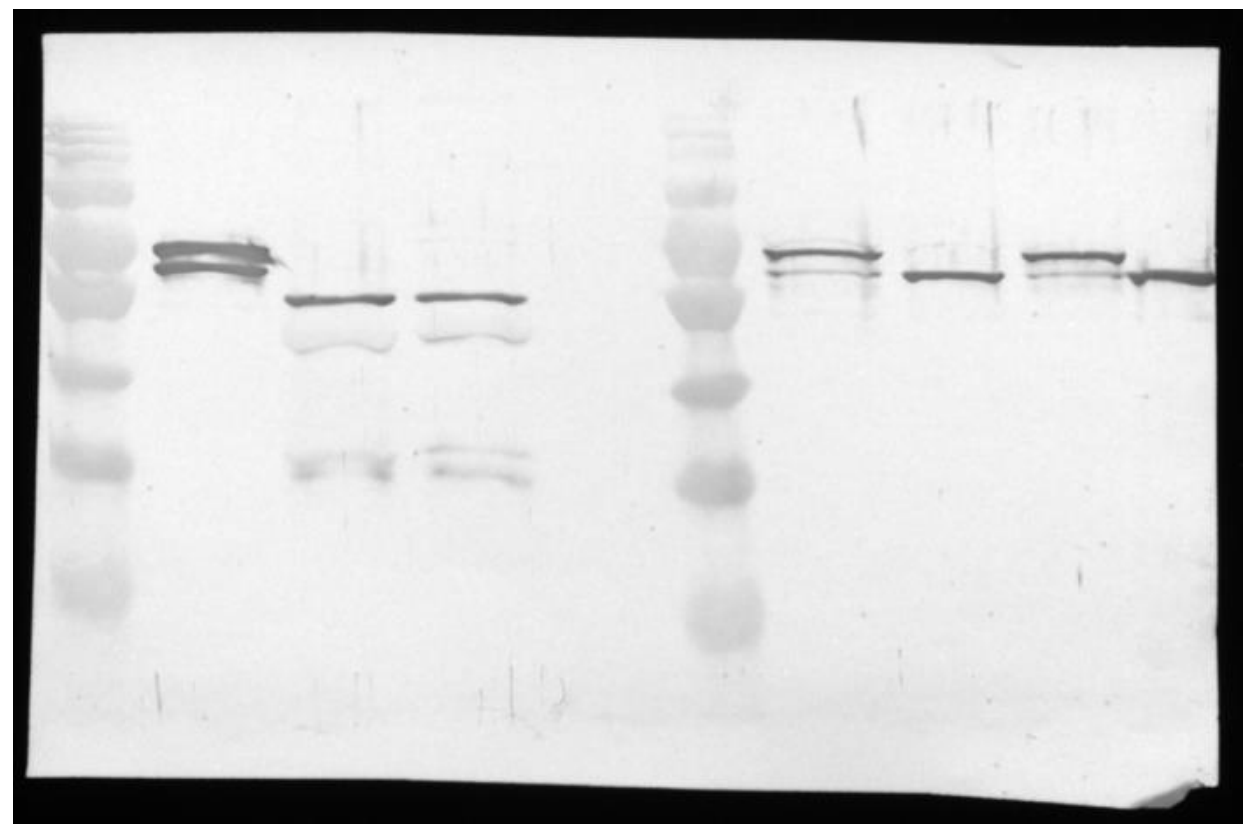

Fig. 6B

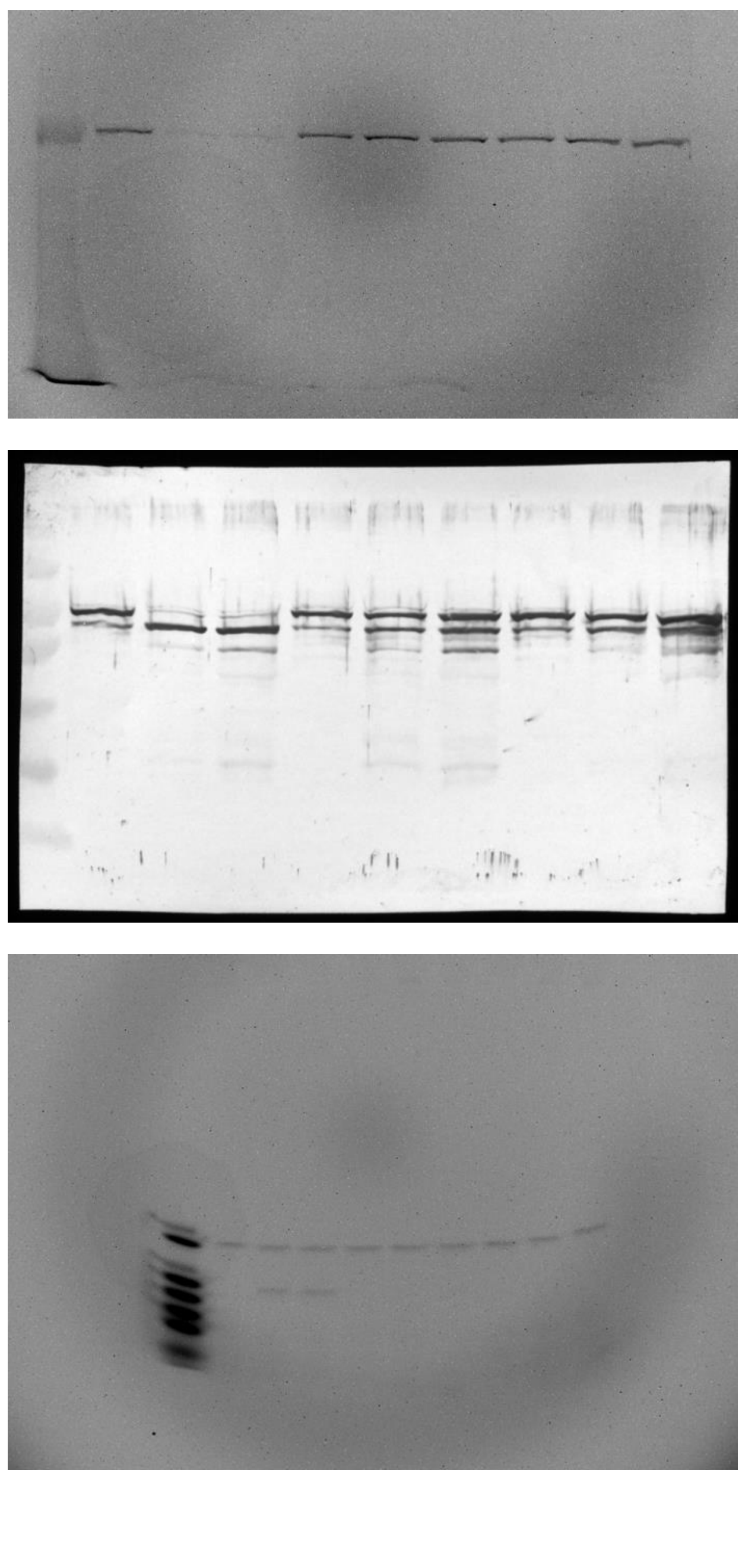

Supplement: Supplementary file 3 [file ppat.1014750.s006.pdf]
