# Supplementary material for: Removal of VirD2 from a synthetic T-DNA complex of Agrobacterium by the host DNA repair protein TDP2
Source: PLoS Pathog. 2026 Oct 26;22(10):e1014750. doi: 10.1371/journal.ppat.1014750 (PMC13727043; doi:10.1371/journal.ppat.1014750)
Supplement: Supplementary file 4 [file ppat.1014750.s003.docx]

**Supplementary Table S1**. Sequences of fluorescently labeled ssDNA segments used for experiments. The two conserved regions of RB T-DNA are indicated in green; the segment that remains attached to VirD2 is indicated in red.

| Total length  (nt) | VirD2-attached segment (nt) | Sequences |
| --- | --- | --- |
| 36^a^ | 9 | CTTAGGTTTACCCGCCAATATATCCTGTCAAACACT-FAM |
| 29^a^ | 9 | TTACCCGCCAATATATCCTGTCAAACACT-FAM |
| 25^a^ | 9 | CCGCCAATATATCCTGTCAAACACT-FAM |
| 19^a^ | 9 | ATATATCCTGTCAAACACT-FAM |
| 15^a^ | 9 | ATCCTGTCAAACACT-FAM |
| 14^a^ | 9 | TCCTGTCAAACACT-FAM |
| 13^a^ | 9 | CCTGTCAAACACT-FAM |
| 9^b^ | 3 | ATCCTGTCA-FAM |
| 8^b^ | 2 | ATCCTGTC-FAM |
| 7^b^ |  | ATCCTGT-FAM |
| 15^c^ | 9 | ATCCTGTCAAACACT-FAM |
| 39^c^ | 33 | ATCCTGTCAAACACTGATAGTTTAAACCGAAGGCGGGAA-FAM |
| 100^c^ | 94 | ATCCTGTCAAACACTGATAGTTTAAACCGAAGGCGGGAAACGACAATCTGATCATGAGCGGAGAATTAAGGGAGTCACGTTATGACCCCCGCCGATGACG-FAM |
| 22^d, e^ | 16 | ATCCTGTCAAACACTGATAGTT-FAM |
| 16^e^ | n.a. | TCAAACACTGATAGTT-FAM |
| 14^e^ | n.a. | AAACACTGATAGTT-FAM |
| 12^e^ | n.a. | ACACTGATAGTT-FAM |
| 10^e^ | n.a. | ACTGATAGTT-FAM |

a, used for the initial experiments and to generate VirD2-ssDNA with decreasing length from the 5’-end (Fig. 1 and 2A)

b, used to generate VirD2-ssDNA with decreasing length from the 3’-end (Fig. 2B)

c, used to generate VirD2-ssDNA with increasing lengths of ssDNA attached segments (Fig. 2C)

d, used to generate VirD2-ssDNA for the experiments with TDP2 (Fig. 3 and 6)

e, used in the size marker mix for the TBE-Urea gel electrophoresis (Fig. 3 and 6)

n.a., not applicable

FAM= 5’-fluorescein phosphoramidite
