# Supplementary material for: Removal of VirD2 from a synthetic T-DNA complex of Agrobacterium by the host DNA repair protein TDP2
Source: PLoS Pathog. 2026 Oct 26;22(10):e1014750. doi: 10.1371/journal.ppat.1014750 (PMC13727043; doi:10.1371/journal.ppat.1014750)
Supplement: Supplementary file 5 [file ppat.1014750.s004.docx]

**Supplementary Table S2**. Sequences of primers used for cloning

| Plasmid | Insert | Primer sequences (Fo= forward, Re= reverse) | |
| --- | --- | --- | --- |
| pEt28a(+) | C58*vir*D2 | Fo  Re | GGACAATTGATGCCCGATCGAGCTCAAGTTATC  GGAGTCGACCTATCTCCTATTTCCCCCACGCC |
| pEt28a(+) | C58virD2-Y29A | Fo  Re | TAGAGCCTCCAACTGATTGATAATTTG  CAAATTATCAATCAGTTGGAGGCTCTA |
| pEt28a(+) | AtTDP2 | Fo  Re | CCGGAATTCATGTCAACAACAATCCGATTTCTGAGAATTG  CGCGTCGACTGATTTTGGCGAAAGGGTTACAAGC |
| pEt28a(+) | AtTDP2-H431N | Fo  Re | GTGTTGCCTAGTGATAATTTCGGTC  GACCGAAATTATCACTAGGCAACAC |
| pCB302T-GFP | AtTDP2 | Fo  Re | CTCAAGCTTCGAATTCTATGTCAACAACAATCCGATTTCTG  TAGACTAGGTGGATCCTCATTTTGGCGAAAGGGTTAC |
